# BM-MSCs overexpressing the *Numb* enhance the therapeutic effect on cholestatic liver fibrosis by inhibiting the ductular reaction

**DOI:** 10.1186/s13287-023-03276-w

**Published:** 2023-03-20

**Authors:** Yan-nan Xu, Wen Xu, Xu Zhang, Dan-yang Wang, Xin-rui Zheng, Wei Liu, Jia-mei Chen, Gao-feng Chen, Cheng-hai Liu, Ping Liu, Yong-ping Mu

**Affiliations:** Shuguang Hospital Affiliated to Shanghai University of Traditional Chinese Medicine (TCM), Institute of Liver Diseases, Shanghai University of TCM, Key Laboratory of Liver and Kidney Disease of the Ministry of Education, Clinical Key Laboratory of TCM of Shanghai, 528, Zhangheng Road, Pudong District, Shanghai, 201203 China

**Keywords:** Cholestasis, Liver fibrosis, *Numb*, BM-MSCs, Notch signaling pathway

## Abstract

**Background:**

Cholestatic liver fibrosis (CLF) is caused by inflammatory destruction of the intrahepatic bile duct and abnormal proliferation of the small bile duct after cholestasis. Activation of the Notch signaling pathway is required for hepatic stem cells to differentiate into cholangiocytes during the pathogenesis of CLF. Our previous research found that the expression of the Numb protein, a negative regulator of Notch signaling, was significantly reduced in the livers of patients with primary biliary cholangitis and CLF rats. However, the relationship between the *Numb* gene and CLF is largely unclear. In this study, we investigated the role of the *Numb* gene in the treatment of bile duct ligation (BDL)-induced CLF.

**Methods:**

In vivo, bone marrow-derived mesenchymal stem cells (BM-MSCs) with *Numb* gene overexpression or knockdown obtained using lentivirus transfection were transplanted into the livers of rats with BDL-induced CLF. The effects of the *Numb* gene on stem cell differentiation and CLF were evaluated by performing histology, tests of liver function, and measurements of liver hydroxyproline, cytokine gene and protein levels. In vitro, the *Numb* gene was overexpressed or knocked down in the WB-F344 cell line by lentivirus transfection, Then, cells were subjected immunofluorescence staining and the detection of mRNA levels of related factors, which provided further evidence supporting the results from in vivo experiments.

**Results:**

BM-MSCs overexpressing the *Numb* gene differentiated into hepatocytes, thereby inhibiting CLF progression. Conversely, BM-MSCs with *Numb* knockdown differentiated into biliary epithelial cells (BECs), thereby promoting the ductular reaction (DR) and the progression of CLF. In addition, we confirmed that knockdown of *Numb* in sodium butyrate-treated WB-F344 cells aggravated WB-F344 cell differentiation into BECs, while overexpression of *Numb* inhibited this process.

**Conclusions:**

The transplantation of BM-MSCs overexpressing *Numb* may be a useful new treatment strategy for CLF.

**Supplementary Information:**

The online version contains supplementary material available at 10.1186/s13287-023-03276-w.

## Background

Cholestasis is a disease characterized by bile formation and excretion disorders caused by various factors. Its typical clinical manifestations are fatigue, itching and jaundice [1]. Primary sclerosing cholangitis (PSC) and, particularly, primary biliary cholangitis (PBC) are the most common cholestatic liver diseases in adults [2]. The pathology of cholestasis is characterized by inflammatory destruction of intrahepatic bile ducts and abnormal proliferation of small bile ducts, which eventually develops into cholestatic liver fibrosis (CLF) and cirrhosis [3]. However, an effective treatment for CLF is still lacking.

The Notch signaling pathway is a highly evolutionarily conserved intercellular signal transduction mechanism. It is necessary for embryonic development, maturation, cell specialization, and maintenance of stem cell characteristics. It plays a critical role in regulating cell fate and maintaining organ morphology [4]. The mammalian Notch signaling pathway consists of four transmembrane receptors (Notch-1/-2/-3/-4) and five ligands (Jagged (JAG)-1/-2 and delta-like (DLL)-1/-3/-4) [5]. Notch receptors are cleaved by *γ*-secretase, and the Notch intracellular domain (NICD) is released. After entering the nucleus, NICD binds to transcription factors, such as recombination signal binding protein Jκ (RBP-Jκ) and CBF1-Suppressor of Hairless-Lag-1 (CSL) and induces the transcriptional activation of Notch target genes such as Hairy and enhancer of split (Hes) and Hes-related with YRPW motif (Hey) [6]. Notch signaling is bidirectional and is a key regulator of the fate of stem cells [7]. At present, it has been recognized that human biliary diseases are closely related to the differentiation of HSCs mediated by the activated Notch signaling [8, 9].

In recent years, the role of Notch signaling in the pathogenesis of liver fibrosis has attracted extensive attention. Aimaiti et al*.* reported that the activation of Notch signaling in hepatic parenchymal cells or nonparenchymal cells activates hepatic stellate cells and promotes the progression of liver fibrosis [10], while the inhibition of JAG1/Notch3 signaling alleviates the activation of hepatic stellate cells and the progression of liver fibrosis [11]. Our previous studies confirmed that the activation of Notch signaling promotes the differentiation of HSCs into biliary epithelial cells (BECs) and the progression of rat CLF induced by bile duct ligation (BDL), whereas blocking Notch signaling inhibits this pathological process [12]. It was also found that the mRNA and protein expression levels of Numb, a negative regulator of Notch signaling, were significantly reduced in the livers of CLF rats [12]. This finding suggested that *Numb* may be involved in the pathological process of CLF.

*Numb* is an evolutionarily highly conserved gene that was first discovered in the precursor cells of the *Drosophila* sensory organ [13], in which Numb determines the fate of stem cells by antagonizing the membrane receptors of the Notch family through asymmetric mitosis [14, 15]. In recent years, many clinical trials of stem cells for liver disease have shown that stem cells might be a potential therapeutic approach [16]. In particular, mesenchymal stem cell-based therapy is currently considered to be an effective treatment strategy for hepatic disorders, such as liver cirrhosis and nonalcoholic fatty liver disease [17]. By comparison, bone marrow mesenchymal stem cells (BM-MSCs, a type of exogenous HSC) are considered to be the most capable stem cell population for liver cell regeneration [18]. Several clinical studies have shown that BM-MSCs are effective and feasible in the treatment of liver cirrhosis [19–21]. However, another study found that BM-MSCs have the potential to differentiate into myofibroblasts after transplantation [22]. Therefore, how to induce the precise differentiation of BM-MSCs into hepatocytes in the cirrhotic liver is a key scientific problem to be solved at present. However, researchers have not clearly determined whether *Numb* alters the differentiation of BM-MSCs or exerts a therapeutic effect on CLF. In the present study, we speculated that Numb may negatively regulate Notch signaling to determine the fate of HSCs and affect the progression or regression of CLF. We prepared a rat model of CLF induced by BDL and transplanted BM-MSCs with *Numb* gene editing into the rat liver to observe their directed differentiation in the liver and their effect on the progression of CLF as a method to confirm this hypothesis. The results are consistent with our hypothesis that Numb determines the fate of BM-MSCs and affects the progression or regression of CLF.

## Methods

### Study design

The goal of this study was to confirm that the high expression of the *Numb* gene in the liver inhibits the activation of the Notch signaling pathway and then inhibits the differentiation of BM-MSCs into BECs and the ductular reaction (DR), which may become a new strategy for the treatment of CLF. First, rat BM-MSCs were isolated and purified, and *Numb* was overexpressed in these cells (BM-MSCs^*Numb*-OE^) by RNA cloning and transfection. Then, BM-MSCs^*Numb*-OE^ were transplanted into the livers of rats with BDL-induced CLF to confirm that BM-MSCs^*Numb*-OE^ differentiate into hepatocytes rather than BECs in the livers of CLF rats, thereby inhibiting the DR and the progression of CLF. Second, rat BM-MSCs were isolated and purified, *Numb* was knocked down (BM-MSC^*Numb*-KD^) by RNA interference, and then these cells were transplanted into the livers of CLF rats to confirm that BM-MSCs^*Numb*-KD^ differentiate into BECs in the livers of rats, which promotes the DR and the progression of CLF. Third, we observed the effect of the *Numb* level on WB-F344 cell differentiation in vitro to further confirm that the *Numb* level determines the fate of HSC differentiation into hepatocytes or BECs by regulating the Notch signaling pathway as a method to provide direct evidence for in vivo experiments.

### Materials

The antibodies used for immunophenotype analysis of the BM-MSCs were mouse monoclonal antibody anti-CD10-fluorescein isothiocyanate (FITC) (MA5-14050; Thermo Fisher Scientific, Waltham, Massachusetts, USA); rabbit polyclonal antibody anti-CD14-FITC (17000-1-AP; Proteintech Group, Chicago, IL, USA); rabbit monoclonal antibody anti-CD34-FITC (ab81289; Abcam, Cambridge, UK); CD45-Alexa Fluor® 488-conjugated antibody (202210; BioLegend, Tokyo, Japan); phycoerythrin (PE)-labeled anti-rat CD29 antibody (562154), CD90-PE antibody (551401), goat anti-rabbit IgG-FITC antibody (554020) and goat anti-rabbit IgG-PE antibody (550083) (BD Biosciences, San Jose, CA, USA).

The types of media used to evaluate the differentiation potential of BM-MSCs were adipogenic differentiation medium (RASMx-90031) and osteogenic differentiation medium (RASMx-90021) for Sprague–Dawley (SD) rat BM-MSCs, both of which were purchased from Cyagen Biosciences Inc. (CA, USA).

The following antibodies were used for immunohistochemistry and immunoblot analysis: mouse monoclonal antibody anti-alpha smooth muscle actin (α-SMA; Clone 1A4; Sigma–Aldrich, St. Louis, MO, USA); rabbit polyclonal antibodies anti-cytokeratin 7 (CK7; 15539-1-AP), CK19 (10712-1-AP) and albumin (Alb; 16475-1-AP); mouse monoclonal antibody anti-Numb (60137-1-Ig) (Proteintech Group Inc., Chicago, IL, USA); rabbit polyclonal antibody anti-Numb (ab220362, Abcam Cambridge, UK); rabbit polyclonal antibody anti-Numb (YT5320, ImmunoWay Biotechnology Company, Newark, DE, USA); mouse monoclonal antibodies anti-hepatocyte nuclear factor 4 alpha (HNF4α; sc-374229) and Sox9 (E-9, sc-166505) (Santa Cruz Biotechnology, Inc., CA, USA); rabbit polyclonal antibody anti-RBP-Jκ (5313; Cell Signaling Technology, Danvers, MA, USA); rabbit polyclonal antibodies anti-Hes1 (ab108937) and EpCam (ab216832) (Abcam, Cambridge, UK); mouse monoclonal antibody anti-glyceraldehyde-3-phosphate dehydrogenase antibody (GAPDH, Chemicon International, Billerica, MA, USA), IRDye 800CW-conjugated donkey anti-mouse IgG (H + L) (LI-COR Bioscience, San Jose, CA, USA) and IRDye 680RD-conjugated donkey anti-rabbit IgG (H + L) (LI-COR Bioscience, San Jose, CA, USA).

### Editing of the *Numb* gene and transplantation of BM-MSCs

#### Isolation and culture of BM-MSCs

BM-MSCs were isolated using the method described by Wang et al. [23]. Briefly, male SD rats were sacrificed under aseptic conditions via cervical dislocation, and the femurs and tibiae were removed. The ends of the femur or tibia were cut open to expose the medullary cavity and repeatedly washed with Dulbecco's modified Eagle’s medium (DMEM, Life Technologies, Gibco, Carlsbad, CA, USA). BM mononuclear cells were isolated using density-gradient centrifugation (Histopaque-1077; Sigma–Aldrich, St. Louis, MO). Mononuclear cells were plated in 75-cm^2^ flasks (Falcon, Franklin Lakes, NJ) with low-glucose DMEM (Gibco, Grand Island, NY) containing 15% fetal bovine serum (FBS; Gibco) and 1% penicillin–streptomycin (Gibco) and cultured at 37 °C in a 5% CO_2_ atmosphere for 24 h. After 24 h, one-half of the volume of the medium was replaced with fresh medium, with the medium was completely replaced at 48 h. After 5–7 days, nonadherent cells were removed by replacing the medium, and adherent cells were cultured for another 2–3 days. Colonized cells were detached with a trypsin/ethylene diamine tetra acetic acid solution (Gibco) and replated in 90-mm Petri dishes. When the cultures approached 70–80% confluence, the cells were serially subcultured through passaging every 3 to 5 days.

#### Immunophenotype, cell cycle analysis and assessment of the differential potential of BM-MSCs

BM-MSC purity was determined by performing immunophenotyping. BM-MSCs were stained with the following antibodies conjugated to FITC or PE: anti-CD10-FITC, anti-CD14-FITC, anti-CD34-FITC, anti-CD45-ALexa Fluor® 488, anti-CD29-PE and anti-CD90-PE. The cells were analyzed using a FACScan flow cytometer (BD Biosciences). Briefly, 5 × 10^5^ cells were resuspended in 0.2 mL of phosphate-buffered saline and incubated with antibodies for 20 min at room temperature (RT). Goat anti-rabbit IgG-FITC and goat anti-rabbit IgG-PE were used as isotype controls. The fluorescence intensity of the cells was evaluated using flow cytometry (BD Biosciences, San Jose, CA, USA) [24–26]. In addition, the cell cycle of BM-MSCs was evaluated using flow cytometry.

Adipogenic and osteogenic induction were performed using a reported method to assess the differentiation potential of BM-MSCs [23]. Briefly, cells were plated on Petri dishes in 15% FBS/DMEM-L. For the induction of adipogenesis, lipogenic induction A solution was added when the cells were 100% confluent. Three days later, the solution was replaced with lipogenic induction B solution (Cyagen Biosciences Inc.), and the solution was replaced with the lipogenic induction A solution after one day. This process was repeated. After 3 weeks of differentiation, the cells were fixed and sliced. Adipogenic differentiation was monitored by observing the red droplets after Oil Red O staining.

For the induction of osteogenic differentiation, the osteoblast induction solution (Cyagen Biosciences Inc.) was added when the cells were 60% confluent, and the solution was changed every 3 days. After 4 weeks of differentiation, the cells were fixed and sliced. The osteogenic-induced culture was analyzed using Alizarin Red staining to visualize calcium deposits.

#### *Numb* cloning and overexpression in BM-MSCs

*Numb* was overexpressed in BM-MSCs (BM-MSCs^*Numb*-OE^) by cloning and transfecting the *Numb* gene. Lentiviral vectors (LV) were labeled with enhanced green fluorescent protein (EGFP). LV-Numb-RNA (titer: 3 × 10^8^ TU/ml, Shanghai Genechem Co., Ltd., Shanghai, China) was transfected into P3 BM-MSCs at a multiplicity of infection (MOI) = 80 with the addition of both polybrene and enhanced infection solution (ENi. S, Shanghai GeneChem Co., Ltd., Shanghai, China). The component sequence of LV-Numb-RNA (20910-4) is Ubi-MCS-3FLAG-SV40-EGFP-IRES-puromycin, and its target sequence is shown in Additional file 1: Text 1. The control BM-MSCs (BM-MSC^overexpression-empty vector^, BM-MSC^OE-EV^) were transfected with CON238, an empty lentivirus vector (titer: 1 × 10^9^ TU/ml, Shanghai Genechem Co., Ltd.), whose component sequence is Ubi-MCS-SV40-EGFP-IRES-puromycin. After transfection for 8–10 h, the medium was replaced with vector-free medium. Next, the cells were transfected with the lentivirus at an MOI = 80.

#### *Numb* knockdown in BM-MSCs

*Numb* was knocked down in BM-MSCs (BM-MSCs^*Numb*-KD^) by RNA interference (RNAi). LV-Numb-RNAi (titer: 6 × 10^8^ TU/ml, Shanghai Genechem Co., Ltd., Shanghai, China) was transfected using the method described above. The component sequence of LV-Numb-RNAi (52,618–1) is hU6-MCS-Ubiquitin-EGFP-IRES-puromycin, and its target sequence is 5′-AAGAGAGGAGATCATGAAACA-3′. Control BM-MSCs (BM-MSCs^knockdown-empty vector^, BM-MSCs^KD-EV^) were transfected with CON077, an empty lentivirus vector (titer: 8 × 10^8^ TU/ml, Shanghai Genechem Co., Ltd.) whose component sequence is hU6-MCS-Ubiquitin-EGFP-IRES-puromycin. After transfection for 8–10 h, the medium was replaced with vector-free medium. Next, the cells were transfected with the lentivirus at an MOI = 80.

#### Animals and experimental protocol

Male SD rats (160–180 g) were purchased from Vital River Laboratory Animal Technology Co., Ltd. (Beijing, China). Animals were maintained in an environment with a constant temperature and supplied with laboratory chow and water ad libitum. All animal experimental protocols were approved by the Animal Research Committee at Shanghai University of Traditional Chinese Medicine (PZSHUTCM18111607), and the study protocols adhere to the ARRIVE guideline.

BDL was performed as previously described [12]. Briefly, 48 rats were randomly divided into the sham group (*n* = 6) and model group (*n* = 42). Model rats were anesthetized with pentobarbital sodium, and laparotomy was performed with sterile technique. The common bile duct and the left and right hepatic ducts were isolated. The left and right hepatic ducts and the hepatic portal and duodenal site of the common bile duct were ligated, and the abdomen was closed. In sham rats, the identical surgery was performed, except that the bile duct was not ligated. After BDL, model rats were randomly divided into the BDL (*n* = 6), BM-MSC (*n* = 6), BM-MSC^OE-EV^ (*n* = 6), BM-MSC^*Numb*-OE^ (*n* = 6), BM-MSC^KD-EV^ (*n* = 6), BM-MSC^*Numb*-KD^ (*n* = 6), and DAPT (*n* = 6, positive control drug) groups, and a single dose of 1 × 10^6^ cells was injected into the spleen of each rat in the corresponding groups. The DAPT group was administered 50 mg/kg DAPT orally once per day for 4 weeks, and sham and BDL rats were administered the same volume of physiological saline. At the end of 4 weeks, all rats were euthanized by administering pentobarbital sodium at a dose of 60 mg/kg, and blood and hepatic tissue samples were obtained.

### Detection of biochemical markers in serum

Serum alanine aminotransferase (ALT), aspartate aminotransferase (AST), total bilirubin (TBil), alkaline phosphatase (ALP), gamma-glutamyltransferase (GGT), total bile acid (TBA), and Alb levels were detected in the clinical laboratory center of Shuguang Hospital affiliated to Shanghai University of TCM.

### Hepatic hydroxyproline (Hyp) content

The Hyp content was determined using the method reported by Jamall et al. [27], with some modifications.

### Histopathological and immunohistochemical analyses

Paraformaldehyde-fixed specimens were cut into 4-μm-thick sections and stained with 0.1% (w/v) Sirius Red or hematoxylin and eosin (H&E). Immunostaining was performed using previously published methods [28]. Briefly, sections were deparaffinized, washed, and preincubated with a blocking solution, followed by an incubation with antibodies against α-SMA (1:200), CK7 (1:100), CK19 (1:100), HNF4α (1:50), OV6 (1:40), Numb (1:50), RBP-Jκ (1:1,000), or Hes1 (1:100). Sections were then incubated with HRP-conjugated secondary antibodies (1:1,000) and washed. The samples were visualized using DAB with hematoxylin counterstaining and imaged with a Leica SCN400 scanner (Leica Microsystems Inc., Concord, ON, Canada).

For immunofluorescence staining, frozen specimens were cut into 7-μm-thick sections and subjected to immunofluorescence staining to detect the coexpression of EGFP (marked LV) and CK7 (1:50), EGFP and CK19 (1:50), EGFP and Alb (1:100), EGFP and HNF4α (1:100), EGFP and CD90 (1:100) and Alb, CK7 and OV6 (1:50), or CK19 and OV6. After an incubation with the primary antibodies, the samples were washed with PBST and incubated with Alexa Fluor 488-conjugated goat anti-mouse IgG (A11001; Invitrogen, Carlsbad, CA, USA) or Alexa Fluor 594-conjugated goat anti-rabbit IgG (AB6939; Abcam, Cambridge, UK) secondary antibodies. The nucleus was stained with 4′,6-diamidino-2-phenylindole (DAPI; 1:1,000), and images were captured using an FV10i confocal laser scanning microscope (Olympus, Japan).

### In vitro experimental protocol

In vitro studies were performed in WB-F344 cell lines, which have morphological and functional characteristics similar to those of freshly isolated hepatic progenitor cells [29].

#### WB-F344 cell culture and treatment

Cells were divided into the normal group (N), sodium butyrate (SB) group (3.75 mM, Sigma, B5887-1G) [30], LV-*Numb* overexpression group (*Numb*-OE), overexpression-empty vector group (OE-EV), LV-*Numb* knockdown group (*Numb*-KD), and knockdown-empty vector group (KD-EV) (*n* = 3 per group).

Cell culture was performed using our previously reported methods [12]. Briefly, cells were cultured at 37 °C in a 5% CO_2_ in air atmosphere with Ham’s F12 medium (Life Technologies) supplemented with 10% fetal calf serum (Life Technologies). Chemically induced differentiation was induced by culturing WB-F344 cells on six-well Permanox Lab-Tek culture slides (NalgeNunc International, Naperville) at a density of 3 × 10^4^ cells/well, starting 24 h after seeding. When the degree of confluence reached 30%, lentiviral transfection was performed (MOI = 50). LV-*Numb*-RNA transfection and LV-*Numb*-RNAi transfection were performed as described above. Then, the culture medium was changed to 10% FBS/DMEM after 6 h of transfection and culture continued until 48 h. SB (3.75 mM) was added to the model group and each intervention group to induce differentiation. The culture medium was exchanged every 2 days, and the cells were collected on the 7th day. The immunofluorescence staining method was the same as described above.

### Real-time PCR (RT–PCR)

The mRNA expression levels of *α-SMA*, collagen I (*Col(1))*, *Col(4)*, tumor necrosis factor alpha (*TNF-α*), transforming growth factor beta 1 (*TGF-β1*), *CK7*, *CK19*, *Numb*, *Hes1*, *RBP-Jκ*, *Notch-1/-2-/3/-4*, *JAG-1/-2*, *DLL-1/-3/-4*, *Sox9*, *EpCam*, ligase Numb protein X1 (*LNX1*), *LNX2* and *ITCH* were assessed using RT–PCR. Total RNA was extracted from frozen hepatic tissues using Isogen (TOYOBO, Kita-ku, Osaka, Japan), and RNA from each sample was reverse transcribed using SuperScript II Reverse Transcriptase (Thermo Fisher Scientific, Waltham, MA, USA). The samples were then analyzed using fluorescence-based RT–PCR and SYBR Green Real-Time PCR Master Mix (TOYOBO) according to the manufacturer’s protocols. Primers and oligonucleotide probes were designed using Primer Express (Takara Chemical) and are listed in Table 1. Each PCR amplification was performed on samples from five rats in both the experimental and control groups. Individual gene expression was normalized to GAPDH. The conditions for the SYBR RT–PCR (Perfect Real Time) were as follows: an initial step of 15 min at 42 °C and 2 min at 95 °C and then 40 amplification cycles of denaturation at 95 °C for 15 s and annealing and extension at 60 °C for 1 min.

**Table 1:** Primer pairs and probes used for real-time PCR

Gene		Primer sequence (5′ → 3′)	Note
*α-SMA*	Forward	AAT GGC TCT GGG CTC TGT AA	SYBR Green
	Reverse	TCT CTT GCT CTG GGC TTC AT	
*Col (1)*	Forward	ACG TCC TGG TGA AGT TGG TC	SYBR Green
	Reverse	TCC AGC AAT ACC CTG AGG TC	
*Col (4)*	Forward	TTT CCA GGG TTA CAA GGT GT	SYBR Green
	Reverse	AGT CCA GGT TCT CCA GCA TC	
*TGF-β1*	Forward	ATT CCT GGC GTT ACC TTG G	SYBR Green
	Reverse	AGC CCT GTA TTC CGT CTC CT	
*TNF-α*	Forward	GAC GTG GAA CTG GCA GAA GAG	SYBR Green
	Reverse	TTG GTG GTT TGT GAG TGT GAG	
*CK7*	Forward	AGG AAC AGA AGT CAG CCA AGA G	SYBR Green
	Reverse	GCA ACA CAA ACT CAT TCT CAG C	
*CK19*	Forward	GAT CTG CGT AGT GTG G-3′	SYBR Green
	Reverse	AAA ACC AAA CTG GGG ATG-3′	
*Numb*	Forward	GCT ACT TTC GAT GCC AGT AGA ACC A	SYBR Green
	Reverse	CTG TTG CCA GGA GCC ACT GA	
*RBP-Jκ*	Forward	TTG CTT ACC TTC AGG CGT GTG	SYBR Green
	Reverse	GCC CAA TGA GTC TGC TGC AA	
*Hes1*	Forward	GAC GGC CAA TTT GCT TTC	SYBR Green
	Reverse	GAC ACT GCG TTA GGA CCC	
*Notch1*	Forward	TGG ATG AGG AAG ACA AGC ATT A	SYBR Green
	Reverse	GAA AAG CCA CCG AGA TAG TCA G	
*Notch2*	Forward	GAG GAA GAA GTG TCT CAA	SYBR Green
	Reverse	GTG GCA TCA GAA ACA TAT G	
*Notch3*	Forward	GAC AAG GAC CAC TCC CAC TACT	SYBR Green
	Reverse	ATC CAC ATC ATC CTC ACA ACT G	
*Notch4*	Forward	TGT CAG GAA CCA GTG TCA GAA C	SYBR Green
	Reverse	CCT GGG CTT CAC ATT CAT CTA T	
*JAG1*	Forward	CCA TCA AGG ATT ATG AGA AC	SYBR Green
	Reverse	TGG TGC TTA TCC ATA TCA	
*JAG2*	Forward	AAA TGA GTG GTC CGT GGC AGA	SYBR Green
	Reverse	TGG TTG GAA GCC TTG TCT GCT	
*DLL1*	Forward	GTG TGC AGA TGG TCC TTG CTT C	SYBR Green
	Reverse	CTG ACA TCG GCA CAG GTA GGA G	
*DLL3*	Forward	CTG AGG TTA CAA GAC GGT GCT	SYBR Green
	Reverse	GTA AAT GGA AGG GGC TGG TAT G	
*DLL4*	Forward	GCA GAA CCA CAC ACT GGA CTA T	SYBR Green
	Reverse	TGG CAC CTT CTC TCC TAA ACT C	
*Sox9*	Forward	GAA AGA CCA CCC CGA TTA CAA G	SYBR Green
	Reverse	AAG ATG GCG TTA GGA GAG ATG TG	
*EpCam*	Forward	TGT GGA CAT AGC TGA TGT GGC TTA C	SYBR Green
	Reverse	CAC CCT CAG GTC CAT GCT CTT A	
*LNX1*	Forward	TGC TGC CAG GAG ACA TCA T	SYBR Green
	Reverse	CAT TGC TTC TGC TAC GGA ACT T	
*LNX2*	Forward	ACA CAG ATT GAG GGT GAA ACT	SYBR Green
	Reverse	GGT CCA CAC AGG AAG AGG T	
*ITCH*	Forward	ATG GGA GAT TTG TCA GTT TGT C	SYBR Green
	Reverse	CAG CGT CAT TCT GTG TAG CA	
*Alb*	Forward	AAG GCA CCC CGA TTA CTC CG	SYBR Green
	Reverse	TGC GAA GTC ACC CAT CAC CG	
*HNF4α*	Forward	CGG GCC ACT GGC AAA CAC	SYBR Green
	Reverse	GTA ATC CTC CAG GCT CAC	
*GAPDH*	Forward	GGC ACA GTC AAG GCT GAG AAT G	SYBR Green
	Reverse	ATG GTG GTG AAG ACG CCA GTA	

### Immunoblot analysis

Liver tissue was lysed in RIPA buffer containing a mixture of protease inhibitors and phosphatase inhibitors and then homogenized in ice-cold water. Protein concentrations were determined using a BCA protein assay kit (Thermo). Total proteins were resolved on SDS–PAGE gels, transferred onto PVDF membranes, and blocked with a 5% (w/v) bovine serum albumin (Gibco) solution. The following dilutions of primary antibodies were used: α-SMA (1:1,000), CK7 (1:1,500), CK19 (1:1,500), Numb (1:300), RBP-Jκ (1:1,000), Hes1 (1:500), Sox9 (1:2,000), EpCam (1:1,000) and GAPDH (1:10,000). The following secondary antibodies were used: IRDye 800CW-conjugated donkey anti-mouse IgG (H + L) (1:10,000) and IRDye 680RD-conjugated donkey anti-rabbit IgG (H + L) (1:1,000). Finally, the data were analyzed using Odyssey 2.1 software.

### Statistical analysis

All data are presented as the means ± SD. Statistical analyses of multiple groups were performed using analysis of variance (ANOVA) with SPSS 24.0 software, and *P* < 0.05 was considered statistically significant.

## Results

### Identification of the purity and proliferation ability of BM-MSCs

**Fig. 1:**
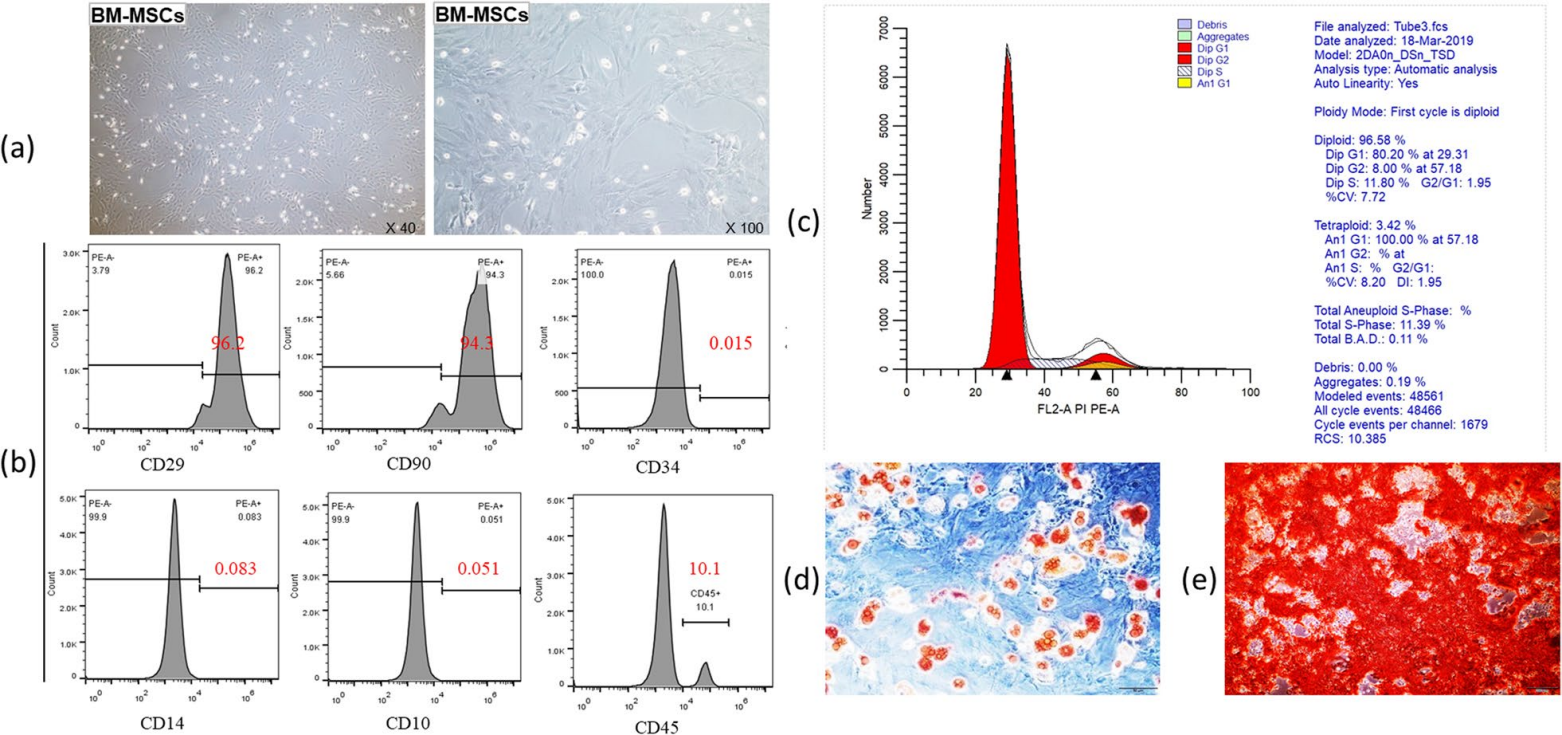
Identification of the purity and proliferation ability of BM-MSCs. **a** BM-MSCs morphology (left: × 40; right: × 100). **b** Cell purity was detected by flow cytometry, and the results showed the following: CD10 (−), CD14 (−), CD29 (+), CD34 (−), CD45 (−) and CD90 (+). **c** The cell cycle was identified by flow cytometry, the result showed that 80.2% of the BM-MSCs were in the G1 phase. **d** Adipogenic induction (× 200). **e** Osteogenic induction (× 200)

When the BM-MSCs were cultured to the third generation, they showed a fusiform and vortex-like morphology (Fig. 1a). BM-MSCs were confirmed by flow cytometry, and showed the following: CD10 (−), CD14 (−), CD29 (+), CD34 (−), CD45 (−) and CD90 (+) (Fig. 1b). Furthermore, the proliferative capacity was evaluated by examining the cell cycle of BM-MSCs using flow cytometry, and the results showed that 80.2% of the BM-MSCs were in G1 phase (Fig. 1c). Additionally, the BM-MSCs showed osteogenic and adipogenic abilities following the differentiation assay. A large number of calcium deposits were noted following osteogenic induction, and a large number of fat droplets were noted following adipogenic induction (Fig. 1d, e). These results demonstrate that the obtained BM-MSCs have a strong differentiation ability.

### BM-MSCs^*Numb*-OE^ transplantation inhibits the progression of CLF induced by BDL

#### BM-MSCs^*Numb*-OE^ transplantation alleviates liver inflammation and fibrosis

**Fig. 2:**
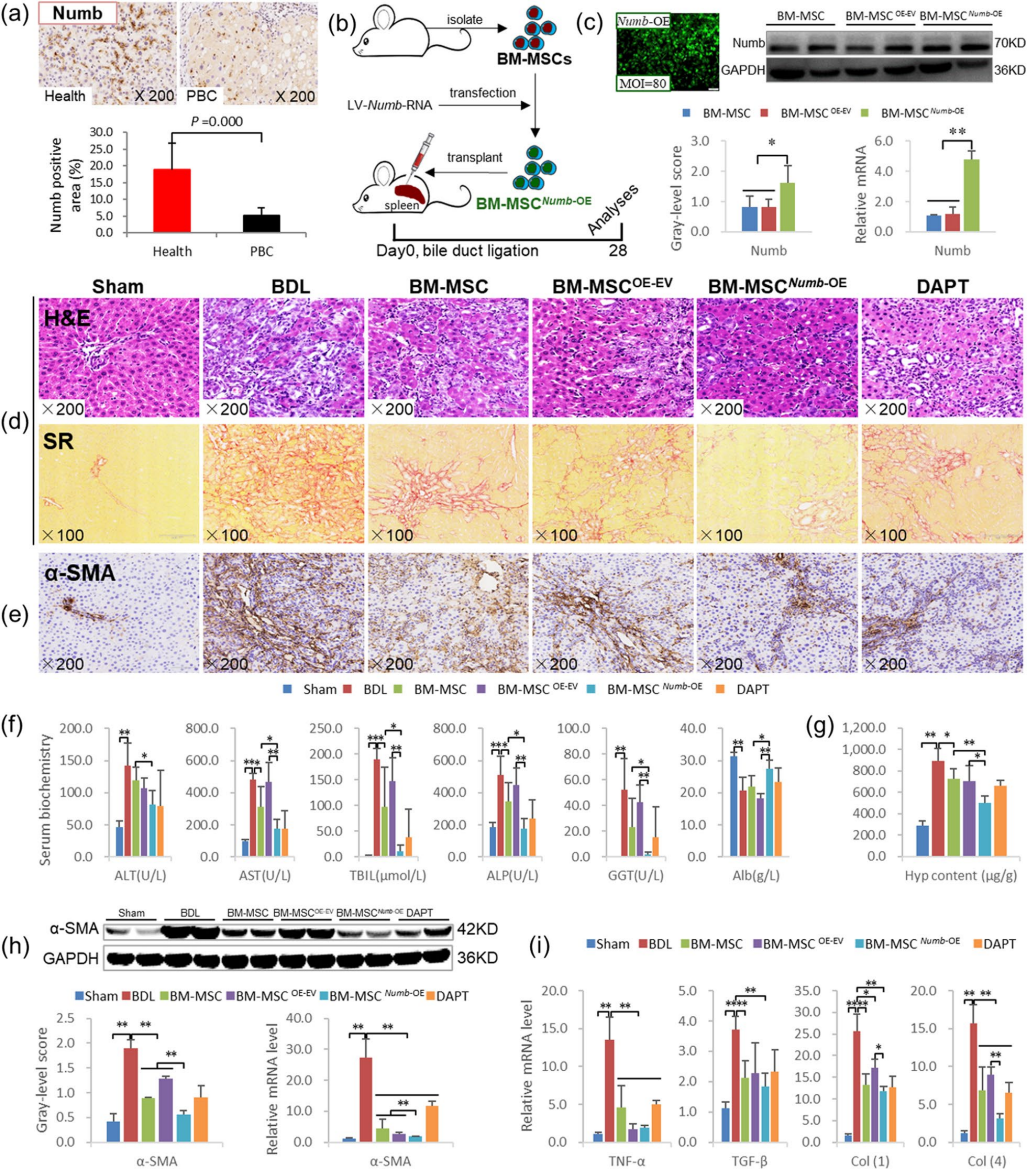
The transplantation of BM-MSCs overexpressing *Numb* inhibits the hepatic inflammatory response and liver fibrosis. **a** Expression of Numb in the livers from a healthy population (*n* = 10) and patients with PBC (*n* = 20): Numb immunostaining (× 200) and its positive area. **b** Experimental flow chart. **c** Lentivirus-transfected BMSCs (× 100) and Numb protein and mRNA expression levels in BM-MSCs overexpressing *Numb* (Full-length blot is presented in Additional file 2: Fig. 1). **d** H&E staining (× 200) and Sirius red collagen staining (× 100). **e** α-SMA immunostaining (× 200). **f** Serum levels of biochemical markers. **g** Hyp content in liver tissue. **h** α-SMA immunoblotting bands, gray-level integration and mRNA expression (*n* = 6/per group) (Full-length blot is presented in Additional file 2: Fig. 2). **i** The mRNA levels of cytokines related to liver fibrosis (*n* = 6/per group). *P < 0.05; **P < 0.01

First, we measured the Numb protein level in the livers of patients with PBC complicated with cirrhosis. Numb was widely expressed in the livers of healthy people, but its expression was clearly decreased in the livers of patients with PBC, as the Numb-positive staining area was reduced by 73% in patients with PBC compared with the healthy population (*P* = 0.000) (Fig. 2a). This result suggests that the loss of Numb may be closely related to the pathogenesis of CLF.

Second, rat BM-MSCs and BM-MSCs^*Numb*-OE^ were transplanted into rats subjected to BDL, and the effect of BM-MSC^*Numb*-OE^ on CLF progression was observed (Fig. 2b; *the experiment was repeated twice*). As shown in Fig. 2c, when the MOI = 80, the transfection rate was greater than 80% and cells maintained a normal morphology. The expression level of the Numb protein was significantly increased in the BM-MSC^*Numb*-OE^ group compared with the BM-MSC^OE-EV^ group (*P* < 0.01), which was 2 times higher than the expression in the BM-MSC^OE-EV^ group, and the level of the *Numb* mRNA was consistent with the protein level (Full-length blot of Numb is presented in Additional file 2: Fig. 1). We performed immunofluorescence costaining for CD90 (a marker of BM-MSCs) and Numb. The results showed that CD90 and Numb were still co-expressed in the BM-MSC^OE-EV^ and BM-MSC^*Numb*-OE^ cells at the end of the 4^th^ week after BDL (Additional file 1: Fig. S1), which confirmed that Numb was located in BM-MSCs in the livers of BDL rats. H&E staining showed that the inflammatory response and bile duct hyperplasia were clearly reduced in the BM-MSC and BM-MSC^OE-EV^ groups compared with the BDL group, and the aforementioned pathological changes were further alleviated in the BM-MSC^*Numb*-OE^ group compared with the BM-MSC^OE-EV^ group (Fig. 2d).

Serum biochemical tests showed significant reductions in AST and ALP activities and the TBil content, revealing that BM-MSCs transplantation significantly improved liver function. Notably, compared with BM-MSCs^OE-EV^ or BM-MSCs transplantation, BM-MSCs^*Numb*-OE^ transplantation further improved serum indicators of liver function, as manifested by significant reductions in AST, ALP and GGT activities and the TBil content and a significant increase in the Alb content (*P* < 0.05 or* P* < 0.01) (Fig. 2f).

Sirius red staining revealed that proliferating BECs were surrounded by abundant collagen in the BDL group; however, collagen deposition was clearly reduced in the BM-MSC and BM-MSC^OE-EV^ groups and was further reduced in the BM-MSC^*Numb*-OE^ group compared with the BM-MSC^OE-EV^ group (Fig. 2d). Consistent with the histopathology, the Hyp content in the liver tissue was significantly increased in the BDL group (*P* < 0.01) but significantly decreased in the BM-MSC and the BM-MSC^OE-EV^ groups compared to the BDL group (*P* < 0.05), and it was further reduced in the BM-MSC^*Numb*-OE^ group compared to the BM-MSC and BM-MSC^OE-EV^ groups (*P* < 0.01 and *P* < 0.05, respectively) (Fig. 2g).

Immunostaining also confirmed that α-SMA expression (a myofibroblast marker) was detected around proliferating BECs in the BDL group; however, its levels were clearly reduced in the BM-MSC and BM-MSC^OE-EV^ groups compared with the BDL group and further reduced in the BM-MSC^*Numb*-OE^ group compared with the BM-MSC^OE-EV^ group (Fig. 2e). Consistent with the immunostaining results, the α-SMA protein and mRNA expression levels were increased significantly in the BDL group (*P* < 0.01), whereas they were significantly reduced in the BM-MSC and BM-MSC^OE-EV^ groups compared to the BDL group (*P* < 0.01) and further reduced in the BM-MSC^*Numb*-OE^ group compared to the BM-MSC and BM-MSC^OE-EV^ groups (*P* < 0.01) (Fig. 2h) (Full-length blot of α-SMA is presented in Additional file 2: Fig. 2).

In addition, the *TGF-β1*, *TNF-α*, *Col(1)*, and *Col(4)* mRNA levels were significantly increased in the liver after BDL (*P* < 0.01), while the *TNF-α*, *Col(1)*, and *Col(4)* mRNA levels were significantly reduced in the BM-MSC and BM-MSC^OE-EV^ groups compared with the BDL group (*P* < 0.05 or *P* < 0.01). Furthermore, the *Col(1)* and *Col(4)* mRNA levels were further reduced in the BM-MSC^*Numb*-OE^ group compared to the BM-MSC^OE-EV^ group (*P* < 0.05 or *P* < 0.01). Although the mRNA levels of *TGF-β1* and *TNF-α* in the BM-MSC^*Numb*-OE^ group were not significantly decreased compared with those in the BM-MSC and BM-MSC^OE-EV^ groups, they were significantly decreased compared with those in the BDL group (*P* < 0.01) (Fig. 2i). Based on these results, BM-MSCs transplantation exerts a good antifibrotic effect, and the intensity of this intervention effect becomes more significant when *Numb* is overexpressed in BM-MSCs.

#### BM-MSCs^*Numb*-OE^ transplantation suppressed the activation of Notch signaling in the livers from CLF rats and differentiation into hepatocytes

**Fig. 3:**
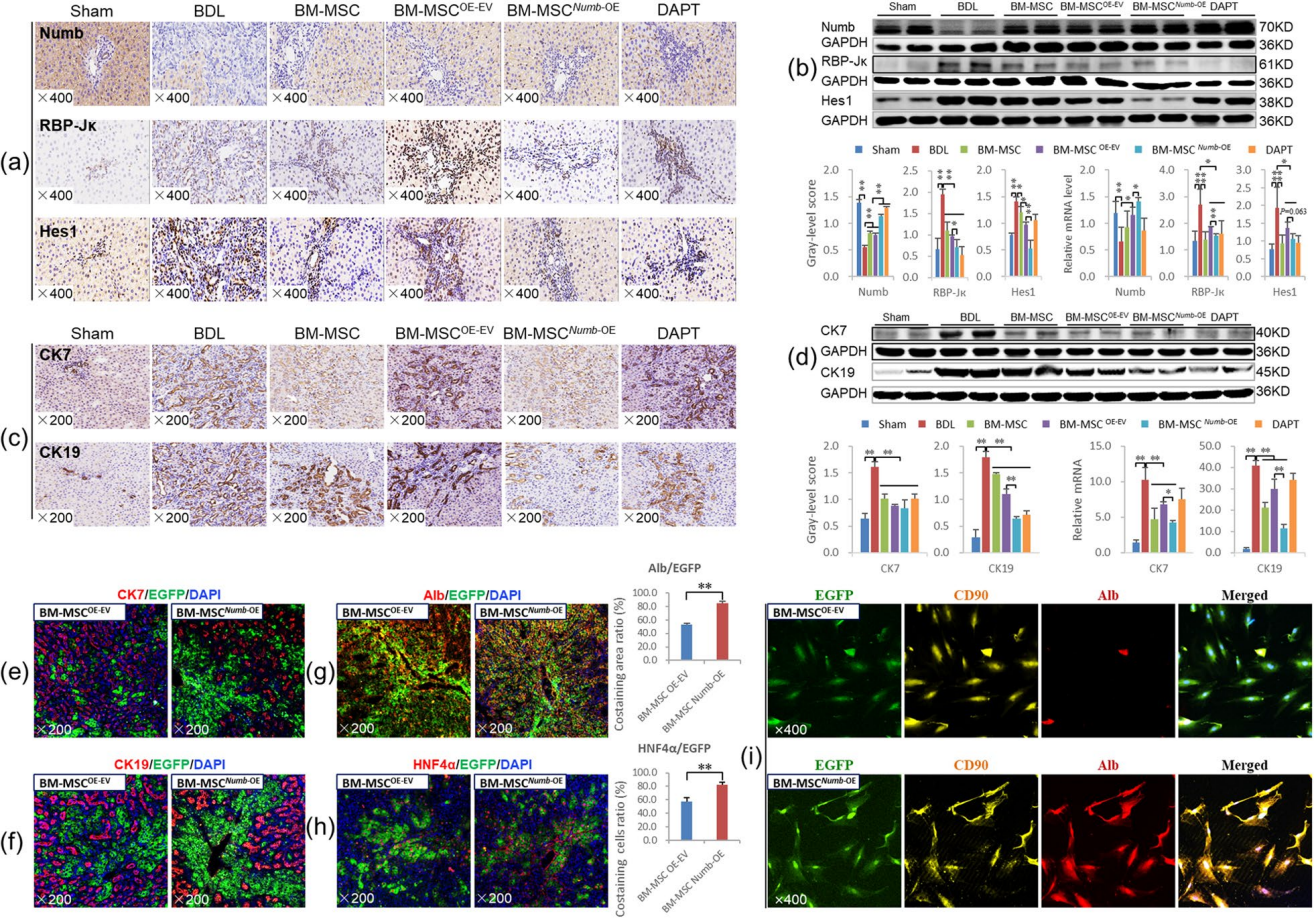
BM-MSCs^*Numb*-OE^ transplantation inhibits the activation of the Notch signaling pathway in the liver. **a** Numb, RBP-Jκ and Hes1 immunostaining (× 400). **b** Protein and mRNA expression levels of Numb, RBP-Jκ, and Hes1 (*n* = 6/per group) (Full-length blots are presented in Additional file 2: Figs. 3–5); **c** CK7 and CK19 immunostaining (× 200). **d** CK7 and CK19 immunoblotting bands, gray-level integration and mRNA expression (*n* = 6/per group) (Full-length blots are presented in Additional file 2: Figs. 6 and 7). **e** CK7/EGFP (labeled BM-MSC^OE-EV^ and BM-MSC^*Numb*-OE^, the same below) immunofluorescence costaining (× 200). **f** CK19/EGFP immunofluorescence costaining (× 200). **g** Alb/EGFP immunofluorescence costaining (× 200), and the costaining area ratio of Alb/EGFP. **h** HNF4α/EGFP immunofluorescence costaining (× 200), and the costaining cells ratio of HNF4α/EGFP. **i** EGFP/CD90/Alb immunofluorescence costaining (× 400). *P < 0.05; **P < 0.01

Immunostaining showed markedly reduced Numb expression in hepatocytes from the BDL group, while the expression levels of RBP-Jκ and Hes1 were markedly increased in the nuclei of proliferating BECs. After transplantation of BM-MSCs, BM-MSCs^OE-EV^ or BM-MSCs^*Numb*-OE^, Numb expression was markedly increased, whereas the expression of RBP-Jκ and Hes1 was decreased, particularly in the BM-MSC^*Numb*-OE^ group (Fig. 3a).

Consistent with the immunostaining results, the Numb protein and mRNA expression levels were significantly reduced in the BDL group (*P* < 0.01), while the RBP-Jκ and Hes1 protein and mRNA levels were significantly increased (*P* < 0.01). Compared with the BDL group, Numb expression was significantly increased in the BM-MSC and BM-MSC^OE-EV^ groups (*P* < 0.01), while RBP-Jκ and Hes1 levels were significantly decreased (*P* < 0.01). In particular, the Numb protein and mRNA levels were further increased, and RBP-Jκ and Hes1 levels were further decreased in the BM-MSC^*Numb*-OE^ group compared to the BM-MSC^OE-EV^ group (*P* < 0.01) (Fig. 3b) (Full-length blots of Numb, RBP-Jκ and Hes1 are presented in Additional file 2: Figs. 3, 4 and 5, respectively). In addition, we evaluated the mRNA expression levels of other related molecules in the Notch signaling pathway. BM-MSCs^*Numb*-OE^ transplantation reduced the expression levels of the *Notch-1/-3/-4* and *JAG2* mRNAs compared with BM-MSCs^OE-EV^ transplantation (*P* < 0.05 or *P* < 0.01) (Additional file 1: Fig. S2). Thus, BM-MSCs transplantation potentially suppresses the activation of Notch signaling, and this effect is more significant after the transplantation of *Numb*-overexpressing BM-MSCs.

Next, the mRNA expression of E3 ubiquitin ligases, including *LNX-1/-2* (promote the proteasome-dependent degradation of Numb) [31] and *ITCH* (promotes the ubiquitination-dependent proteasomal degradation of the NICD) [32], was examined. *LNX1* expression was increased significantly, and *LNX2* and *ITCH* levels were decreased significantly in the BDL group compared with the sham group (*P* < 0.01). However, compared to the levels in the BDL group, *LNX1* expression was decreased significantly in the BM-MSC^OE-EV^ group (*P* < 0.05). Only *ITCH* expression was increased significantly in the BM-MSC^*Numb*-OE^ group compared to the BM-MSC^OE-EV^ group (*P* < 0.05) (Additional file 1: Fig. S3). Based on these results, BM-MSCs^*Numb*-OE^ transplantation may increase the ubiquitination of Notch due to the increase in the Numb level [33], which leads to the suppression of Notch signaling in BM-MSCs. Then, the expression levels of other Notch signaling molecules upstream of Numb are decreased, which reduces the differentiation of BM-MSCs into BECs and inhibits CLF progression.

CK7 is considered a marker of hepatic progenitor cells [34], and CK19 is a recognized marker of BECs [35]. As shown in Fig. 3c, immunostaining revealed that CK7 and CK19 were widely expressed in proliferating BECs in the BDL group, while their expression was clearly reduced in the BM-MSC, BM-MSC^OE-EV^ and BM-MSC^*Numb*-OE^ groups, particularly in the BM-MSC^*Numb*-OE^ group. Consistent with the immunostaining data, the expression levels of the CK7 and CK19 proteins were increased significantly in the BDL group (*P* < 0.01), whereas they were significantly reduced in the BM-MSC and BM-MSC^OE-EV^ groups compared to the BDL group (*P* < 0.01). Additionally, the CK19 protein level was further reduced in the BM-MSC^*Numb*-OE^ group compared with the BM-MSC^OE-EV^ group (*P* < 0.01) (Fig. 3d) (Full-length blots of CK7 and CK19 are presented in Additional file 2: Figs. 6 and 7, respectively). The *CK7* and *CK19* mRNA expression levels were consistent with their protein expression levels (Fig. 3d). This finding suggests that BM-MSC^*Numb*-OE^ transplantation inhibits the DR in CLF rats.

To determine the differentiation direction of BM-MSCs in CLF liver, we labeled cells with EGFP (labeled lentivirus, showing traces of BM-MSC^OE-EV^ and BM-MSC^*Numb*-OE^) and costained them with antibodies against CK7, CK19, Alb (synthesized by mature hepatocytes) or HNF4α (a mature hepatocyte marker) to evaluate the oriented differentiation of BM-MSCs^*Numb*-OE^ in the liver. As shown in Fig. 3e–h, very little coexpression of EGFP/CK7 or EGFP/CK19 was detected in the BM-MSC^*Numb*-OE^ group, while EGFP/Alb and EGFP/HNF4α were widely coexpressed in hepatocytes. To evaluate the ability of BM-MSCs^OE-EV^ or BM-MSCs^*Numb*-OE^ to differentiate into hepatocytes, we analyzed the positive area ratio of EGFP/Alb immunofluorescence costaining and the positive cell ratio of EGFP/HNF4α immunofluorescence costaining. The results showed that the costaining area ratio of EGFP/Alb in the BM-MSC^*Numb*-OE^ group was 1.6 times that in the BM-MSCs^OE-EV^ group (84.25% *vs.* 57.70%, *P* = 0.000) (Fig. 3g histogram), and the costaining cell ratio of EGFP/HNF4α in the BM-MSC^*Numb*-OE^ group was 1.4 times that in the BM-MSCs^OE-EV^ group (82.31% *vs.* 57.75%, *P* = 0.003) (Fig. 3h histogram). In addition, we observed the ability of BM-MSCs^*Numb*-OE^ to differentiate into hepatocytes in vitro. We performed immunofluorescence staining for EGFP (labeled BM-MSC^OE-EV^ and BM-MSC^*Numb*-OE^), CD90 (a marker of BM-MSCs) and Alb (a marker of hepatocytes), and the results showed that there was extensive coexpression of EGFP/CD90/Alb in the BM-MSC^*Numb*-OE^ cells compared with BM-MSC^OE-EV^ cells on the 6^th^ day of cultivation (Fig. 3i). Thus, BM-MSCs overexpressing *Numb* differentiate into hepatocytes rather than BECs in the liver of CLF rats.

### BM-MSCs^*Numb*-KD^ transplantation promotes the progression of CLF induced by BDL

#### BM-MSCs^*Numb*-KD^ transplantation promotes liver inflammation and fibrosis

**Fig. 4:**
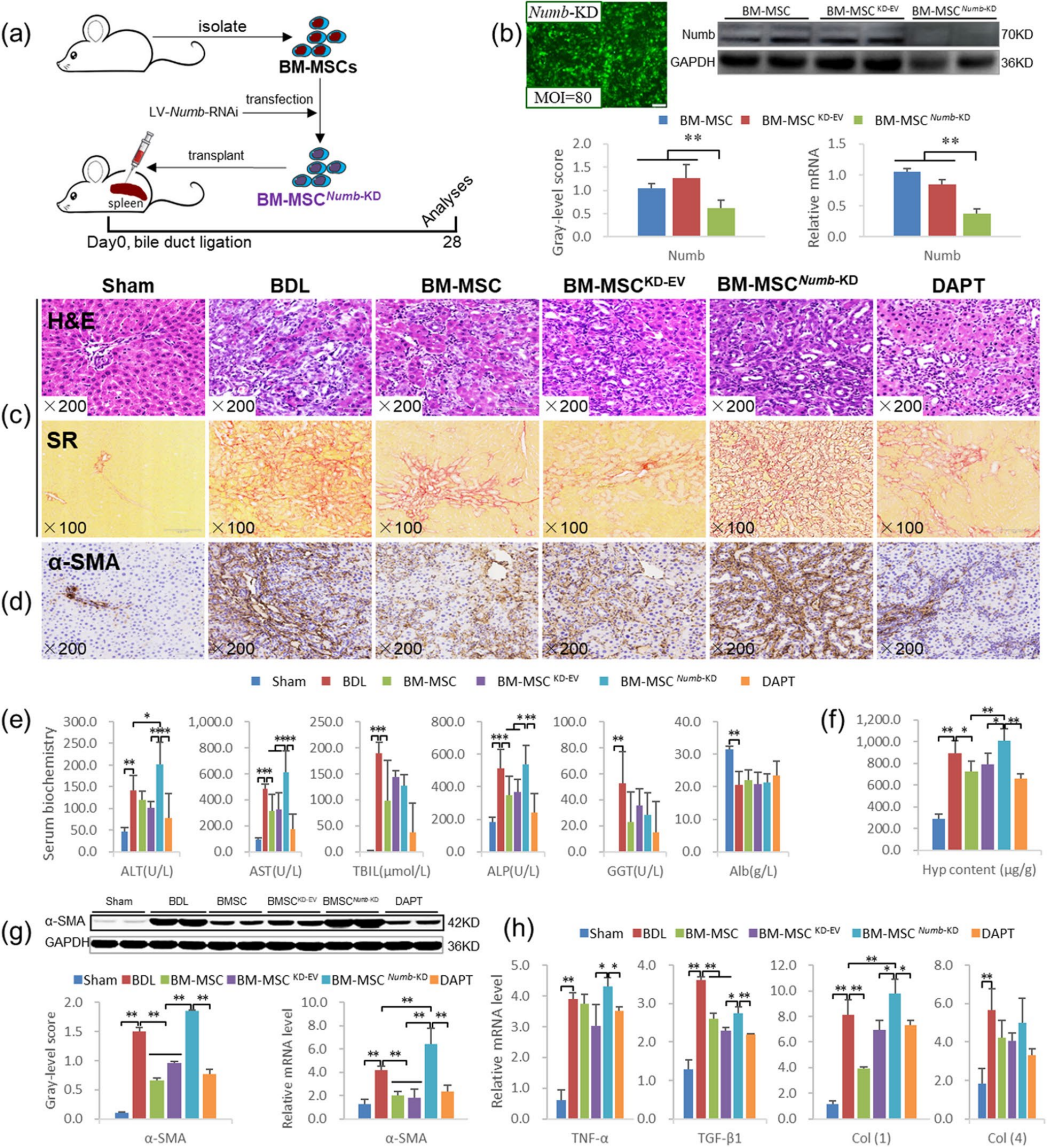
BM-MSCs^*Numb*-KD^ transplantation promotes the hepatic inflammatory response and liver fibrosis. **a** Experimental flow chart. **b** Lentivirus-transfected BMSCs (× 100) and Numb protein and mRNA expression levels in BM-MSCs with knockdown of *Numb* (Full-length blot is presented in Additional file 2: Fig. 8). **c** H&E staining (× 200) and Sirius red collagen staining (× 100). **d** α-SMA immunostaining (× 200). **e** Serum levels of biochemical markers and **f** the Hyp content in liver tissues. **g** α-SMA immunoblotting bands, gray-level integration and mRNA expression (*n* = 6/per group) (Full-length blot is presented in Additional file 2: Fig. 9); **h** The mRNA expression levels of *TGF-β1*, *TNF-α*, *Col(1)*, and *Col(4)*. *P < 0.05; **P < 0.01

We knocked down *Numb* in BM-MSCs by RNA interference (BM-MSC^*Numb*-KD^), injected the cells into the rat spleen at the same time as BDL, and obtained samples at the end of 4 w to determine whether the deletion of *Numb* in BM-MSCs promotes the progression of CLF (Fig. 4a, *the experiment was repeated twice*). As shown in Fig. 4b, when the MOI = 80, the transfection rate was greater than 80% and the cells maintained a normal morphology. The expression level of the Numb protein was significantly decreased in the BM-MSC^*Numb*-KD^ group compared with the BM-MSC^KD-EV^ group (*P* < 0.01) to approximately 51.2% of that in the BM-MSC^KD-EV^ group (Full-length blot of Numb is presented in Additional file 2: Fig. 8), and the level of the *Numb* mRNA was consistent with the protein level. As mentioned above, BM-MSCs and BM-MSCs^KD-EV^ transplantation alleviated liver inflammation, the DR, and collagen deposition; improved serum biochemical indexes; and decreased the Hyp content and the expression levels of proteins related to liver fibrosis, including α-SMA, TGF-β1 and Col(1). However, when BM-MSCs^*Numb*-KD^ were transplanted, liver inflammation, the DR and collagen deposition were markedly increased compared with those in the BM-MSC^KD-EV^ group (Fig. 4c). In addition, serum ALT, AST and ALP activities (Fig. 4e), the Hyp content (Fig. 4f), α-SMA expression (Fig. 4d, g) (Full-length blot of α-SMA is presented in Additional file 2: Fig. 9) and the mRNA expression levels of proteins related to liver fibrosis, including *TNF-α*, *TGF-β1* and *Col(1)* (Fig. 4h), were increased significantly in the BM-MSC^*Numb*-KD^ group compared to the BM-MSC^KD-EV^ group (*P* < 0.05 or *P* < 0.01). These results suggested that BM-MSCs^*Numb*-KD^ transplantation aggravates the liver inflammatory response and hepatic stellate cell activation and therefore promotes the progression of CLF induced by BDL.

#### BM-MSCs^*Numb*-KD^ transplantation activates Notch signaling in the livers of CLF rats and promotes differentiation into BECs

**Fig. 5:**
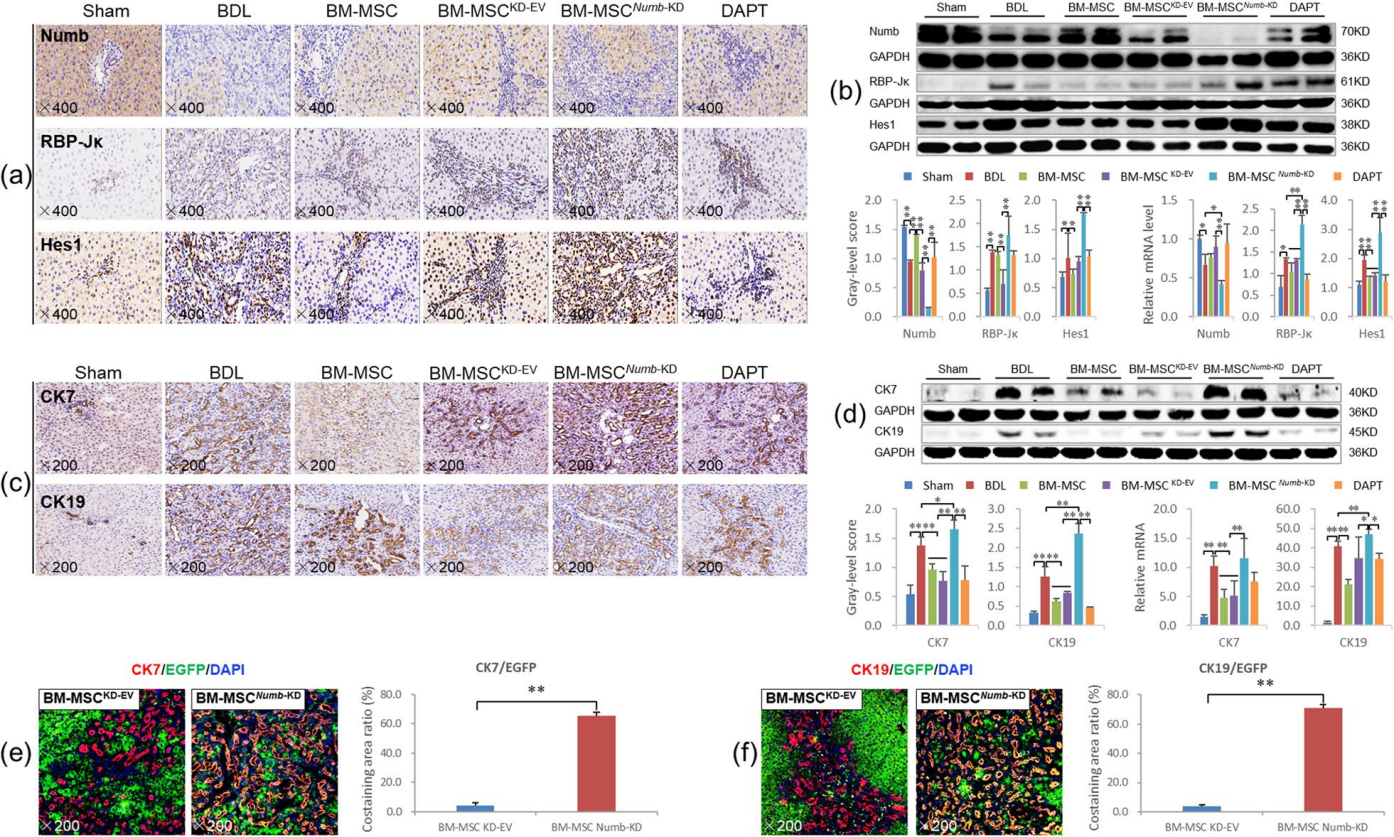
BM-MSC^*Numb*-KD^ transplantation activates Notch signaling in the livers of CLF rats and induces the differentiation of these cells into BECs. **a** Numb, RBP-Jκ and Hes1 immunostaining (× 400). **b** Protein and mRNA expression levels of Numb, RBP-Jκ, and Hes1 (*n* = 6/per group) (Full-length blots are presented in Additional file 2: Figs. 10–12). **c** CK7 and CK19 immunostaining (× 200). **d** Protein and mRNA expression levels of CK7 and CK19 (Full-length blots are presented in Additional file 2: Figs. 13 and 14). **e** CK7/EGFP (labeling BM-MSC^KD-EV^ and BM-MSC^*Numb*-KD^) immunofluorescence costaining (× 200), and the costaining area ratio of CK7/EGFP. **f** CK19/EGFP immunofluorescence costaining (× 200), and the costaining area ratio of CK19/EGFP. *P < 0.05; **P < 0.01

As mentioned above, transplantation of BM-MSCs significantly inhibits the activation of the Notch signaling pathway in the livers of CLF rats. However, when BMSCs lacking *Numb* were transplanted, Notch signaling was significantly activated, as confirmed by the protein and mRNA expression levels of Numb, RBP-Jκ and Hes1; namely, the expression of Numb was decreased significantly and the expression levels of RBP-Jκ and Hes1 were increased significantly compared to those in the BM-MSC^KD-EV^ group (*P* < 0.01). In addition, the *Numb* mRNA level in the BM-MSC^*Numb*-KD^ group was significantly lower than that in the BDL group (*P* < 0.05), while significantly higher levels of the *RBP-Jκ* and *Hes1* mRNAs were detected than those in the BDL group (*P* < 0.01) (Fig. 5a, b) (Full-length blots of Numb, RBP-Jκ and Hes1 are presented in Additional file 2: Figs. 10, 11 and 12, respectively). Furthermore, the mRNA expression levels of other related molecules in the Notch pathway, including *Notch-2/-3/-4*, *JAG-1/-2*, and *DLL-1/-4*, were also significantly increased in the BM-MSC^*Numb*-KD^ group compared to the BM-MSC^KD-EV^ group (*P* < 0.05 or *P* < 0.01) (Additional file 1: Fig. S4). In addition, we detected the mRNA expression levels of *LNX-1/-2* and *ITCH*, and only *ITCH* expression was decreased significantly in the BM-MSC^*Numb*-KD^ group compared to the BM-MSC^KD-EV^ group (*P* < 0.05) (Additional file 1: Fig. S5). These results suggest that BM-MSCs^*Numb*-KD^ transplantation may reduce the ubiquitination of Notch due to the decrease in the Numb level [33], which leads to the activation of Notch signaling in BM-MSCs, thus promotes the differentiation of BM-MSCs into BECs and aggravates CLF progression.

As mentioned above, BM-MSCs transplantation significantly suppressed the DR in CLF livers. However, BM-MSCs^*Numb*-KD^ transplantation significantly promoted the DR compared with BDL and BM-MSCs^KD-EV^ transplantation, as confirmed by the protein and mRNA expression levels of CK7 and CK19 (Fig. 5c, d) (Full-length blots of CK7 and CK19 are presented in Additional file 2: Figs. 13 and 14, respectively).

We evaluated the oriented differentiation of BM-MSCs^*Numb*-KD^ in the livers of CLF rats by observing cells with coexpression of EGFP (labeled lentivirus, showing traces of BM-MSC^LV-EV^ and BM-MSC^*Numb*-KD^) and CK7 or CK19. The results showed very little coexpression of EGFP with CK7 or EGFP with CK19 in the BM-MSC^KD-EV^ group, but extensive coexpression of EGFP with CK7 and CK19 was observed in the BM-MSC^*Numb*-KD^ group (Fig. 5e, f). In addition, we analyzed the positive area ratio of CK7/EGFP and CK19/EGFP immunofluorescence costaining. The results showed that the costaining area ratio of EGFP/CK7 in the BM-MSC^*Numb*-KD^ group was 14.8 times that in the BM-MSCs^KD-EV^ group (65.73% *vs.* 4.44%, *P* = 0.000) (Fig. 5e histogram), and the costaining area ratio of EGFP/CK19 in the BM-MSC^*Numb*-KD^ group was 18.5 times that in the BM-MSCs^KD-EV^ group (71.11% *vs.* 3.84%, *P* = 0.000) (Fig. 5f histogram). The aforementioned results clearly indicate that BM-MSCs lacking *Numb* differentiated into BECs in the livers of CLF rats and promoted the DR.

### The *Numb* level determines the fate of HSCs in vitro

**Fig. 6:**
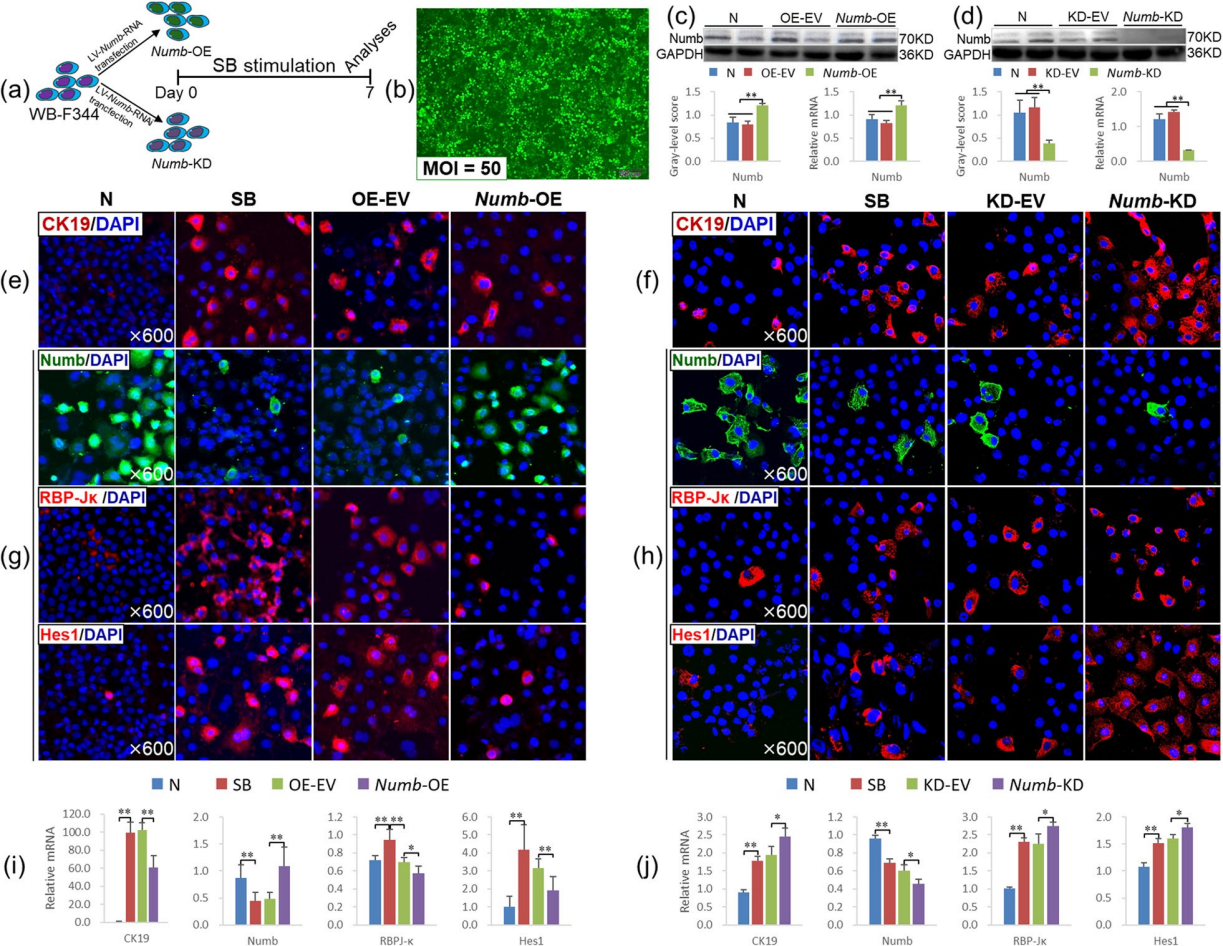
Effect of *Numb* expression on the differentiation of WB-F344 cells. **a** Experimental flow chart. **b** Cell morphology and GFP expression after lentivirus transfection for 72 h (× 100). **c**, **d** Numb protein and mRNA levels (Full-length blots are presented in Additional file 2: Figs. 15 and 16). **e**, **f** CK19 immunofluorescence staining (× 600). **g**, **h** Immunofluorescence staining for Numb, RBP-Jκ and Hes1 (× 600). **i**, **j** The mRNA levels of *CK19*, *Numb*, *RBP-Jκ* and *Hes1*. *P < 0.05; **P < 0.01

We overexpressed or knocked down *Numb* in WB-F344 cells (*Numb*-OE or *Numb*-KD) and stimulated them with SB to further clarify the regulatory effect of *Numb* on the differentiation fate of HSCs (Fig. 6a). When the lentivirus was added at an MOI = 50, the transfection rate was greater than 80%, and the cell morphology was normal (Fig. 6b). In addition, Numb protein expression was increased significantly in the *Numb*-OE group compared to the OE-EV group (*P* < 0.01), whereas it was decreased significantly in the *Numb*-KD group compared to the KD-EV group (*P* < 0.01), and the *Numb* mRNA level was consistent with the corresponding protein level (Fig. 6c, d) (Full-length blots of Numb are presented in Additional file 2: Figs. 15 and 16).

Immunostaining showed a clear increase in CK19 expression in the SB, OE-EV and KD-EV groups, but its expression was decreased in the *Numb*-OE group and further increased in the *Numb*-KD group (Fig. 6e, f). The expression level of the *CK19* mRNA was consistent with the immunostaining results (Fig. 6i, j).

In addition, immunostaining showed a clear decrease in Numb expression, and the expression of RBP-Jκ and Hes1 was increased in the SB, KD-EV and OE-EV groups. Compared with that in the OE-EV group, Numb expression was increased, and the expression of RBP-Jκ and Hes1 was decreased in the *Numb*-OE group (Fig. 6g). Conversely, the expression of Numb was further decreased, and RBP-Jκ and Hes1 levels were further increased in WB-F344 cells lacking *Numb* (Fig. 6h).

Consistent with the immunostaining results, *Numb* mRNA levels were decreased significantly (*P* < 0.01), and those of *RBP-Jκ* and *Hes1* were increased significantly in the KD-EV and OE-EV groups (*P* < 0.01). However, the *Numb* mRNA levels were increased and those of *RBP-Jκ* and *Hes1* were significantly decreased in the *Numb*-OE group compared to the OE-EV group (*P* < 0.05 or *P* < 0.01) (Fig. 6i). Conversely, the *Numb* mRNA expression levels were further reduced, and those of *RBP-Jκ* and *Hes1* were further increased in the *Numb*-KD group compared to the KD-EV group (*P* < 0.05) (Fig. 6j).

Therefore, WB-F344 cells lacking *Numb* differentiate into BECs when stimulated with SB; conversely, the overexpression of *Numb* in WB-F344 cells suppresses this pathological process.

## Discussion

The liver is a very complex organ that is susceptible to multiple types of damage and dysfunction [36]. Biliary proliferation, also known as the DR, occurs when BECs are stimulated by persistent inflammation [37], and HSCs are an important participant in the DR [38, 39]. Proliferating BECs secrete a variety of profibrotic cytokines, such as TNF-α, TGF-β1, platelet-derived growth factor (PDGF), interleukin (IL)-1, -6, -8, and monocyte chemoattractant protein 1 (MCP1). They synergistically promote the activation of fibroblasts and hepatic stellate cells around portal veins into myofibroblasts, synthesize a large amount of extracellular matrix, and promote the occurrence and development of liver fibrosis [40]. Therefore, inhibition of the abnormal activation and proliferation of BECs may partially or even completely reverse CLF [33].

Activation of Notch signaling plays a critical role in the DR [41]. In our previous study, we confirmed that blocking the Notch signaling pathway significantly inhibits the differentiation of HSCs into BECs and the progression of CLF induced by BDL, and we found that the Numb mRNA and protein levels gradually decrease with CLF progression [12]. In addition, the expression of Numb in the livers of patients with PBC was only 26.95% of the level in healthy subjects. However, the role of Numb in the occurrence and treatment of CLF has not been reported. This study is the first to focus on the effect of *Numb* on CLF.

### The *Numb* level in HSCs determines their fate in the livers of CLF rats

Numb negatively regulates Notch signaling and antagonizes the membrane receptors of the Notch family through asymmetric mitosis, which is an important determinant of cell fate [14]. In recent years, Numb has attracted extensive attention in tumor therapy; for example, the loss of *Numb* expression may increase Notch signaling activity in breast cancer cells [42]. *Numb* may be a therapeutic target for prostate cancer by inhibiting the activation of Notch signaling [43]. Moreover, the level of *Numb* is significantly decreased in human hepatocellular carcinoma, and miR-148a upregulates *Numb* expression to inhibit Notch signaling, thereby inhibiting hepatocellular carcinoma progression [44]. In the field of chronic liver disease, Numb may act as a "switch" of the Wnt-Notch signaling pathway, which determines the differentiation of HSCs into bile duct cells (activation of Notch signaling) or hepatocytes (activation of classical Wnt signaling) [45]. This finding highlights the importance of *Numb* in regulating the differentiation of HSCs.

Bone marrow is an important source of exogenous HSCs [46]. In a rat model of acute liver injury, transplantation of BM-MSCs significantly reduced the levels of liver injury markers [47]. In patients with alcoholic cirrhosis, transplantation of autologous BM-MSCs safely improved histologic fibrosis and liver function [20].

In this study, we first observed the effects of transplantation of BM-MSCs lacking or overexpressing *Numb* on CLF to directly observe the role of *Numb* expressed in HSCs in CLF pathogenesis. The results clearly showed that BM-MSCs^*Numb*-OE^ transplantation effectively inhibits hepatic inflammation, the DR and CLF progression. The main mechanism is that BM-MSCs overexpressing Numb mainly differentiated into hepatocytes, as evidenced by the significantly increased levels of the Alb and HNF4α mRNAs and proteins in the liver and serum Alb content; immunofluorescence costaining also clearly suggested that BM-MSCs^*Numb*-OE^ differentiated into hepatocytes and promoted the repair of liver injury.

In contrast, BM-MSCs^*Numb*-KD^ transplantation significantly promotes the inflammatory response in the liver, hepatic stellate cell activation and CLF progression. The main mechanism is that the Notch signaling is activated in BM-MSCs that then differentiate into BECs due to the weakened negative regulation of Notch signaling after *Numb* loss, as manifested by CK7/EGFP, CK19/EGFP are widely coexpressed in proliferating BECs, and the DR is enhanced, thus promoting the progression of CLF. In addition, in vitro experiments confirmed that WB-F344 cells lacking *Numb* differentiate into a bile duct cell phenotype.

### The role of *Numb* in determining the fate of stem cells depends on its negative regulation of the Notch signaling pathway

The Notch signaling pathway is bidirectional and plays an important role in regulating the fate of stem cells [7]. For example, intestinal stem cells differentiate into intestinal cells and endocrine cells in adult fruit flies and mice. Inhibition of Notch signaling leads to the differentiation of intestinal stem cells into intestinal endocrine cells, while activation of Notch signaling promotes differentiation into intestinal cells [48]. In human diseases, inhibition of Notch signaling suppresses the self-renewal ability of lung adenocarcinoma stem cells and promotes their re-entry into asymmetric division [49]. Therefore, we speculate that Notch signaling may also regulate the mitotic state or pluripotency of stem cells in other organs.

As shown in our previous study, Notch signaling in the liver is gradually activated with the progression of CLF, while this pathological change is blocked by DAPT (a *γ*-secretase inhibitor) [12]. Thus, the inhibition of Notch signaling may be crucial for the treatment of CLF. In the present study, we first tested the effects of BM-MSCs with different *Numb* levels on hepatic Notch signaling after transplantation to clarify the mechanism by which changes in the *Numb* level in HSCs affect their differentiation. Consistent with our hypothesis, when *Numb* was deleted from BM-MSCs, the expression of Notch signaling factors downstream of RBP-Jκ and Hes1 was significantly increased. In addition, the mRNA expression levels of other components of Notch signaling, including *Notch-2/-3/-4*, *JAG-1/-2* and *DLL-1/-4,* were also increased significantly and jointly promoted Notch signaling activation. When BM-MSCs overexpressed *Numb*, the expression levels of RBP-Jκ and Hes1 were significantly reduced. In addition, the mRNA expression levels of other components of Notch signaling, including *Notch-2/-3/-4*, *JAG-1/-2* and *DLL-3,* were also significantly reduced and thus jointly inhibited the activation of Notch signaling. These results were also confirmed in vitro. Based on these results, the effect of Numb on determining the fate of HSCs depends on its negative regulation of Notch signaling, and this effect may be related to Numb-mediated promotion of Notch ubiquitination in rats with CLF.

In recent years, a large number of studies have shown that the biological antagonism between Numb and Notch controls the balance of stem cell proliferation and differentiation in development and homeostasis, and this biological antagonism depends on a series of ubiquitination processes. Mammalian Numb is clearly the substrate of E3 ubiquitin LNX, and wild-type LNX causes proteasome-dependent Numb degradation, which enhances the activity of Notch signaling [50]. On the other hand, Numb works with ITCH, another E3 ubiquitin ligase, in the cytoplasm to promote the ubiquitination of Notch in the cell membrane, thereby promoting the degradation of the NICD and avoiding its nuclear translocation and downstream target gene activation [51]. Thus, the balance of Numb–Notch ubiquitination may play an important role in maintaining liver homeostasis. In this study, after BM-MSCs^*Numb*-KD^ transplantation, the level of the *LNX* mRNA did not change in the liver but that the level of *ITCH* mRNA was significantly reduced, suggesting that BM-MSCs^*Numb*-KD^ transplantation attenuated the ubiquitination-mediated degradation of Notch. In contrast, after BM-MSCs^*Numb*-OE^ transplantation, the level of *LNX* mRNA did not change, but the level of *ITCH* mRNA was significantly increased, suggesting that BM-MSCs^*Numb*-OE^ transplantation promoted Notch ubiquitination. However, relevant evidence on methods to regulate the balance of Numb–Notch ubiquitination after hepatic *Numb* supplementation is still lacking.

In summary, *Numb* plays an important role in the occurrence and repair of CLF, and its key mechanism is to regulate Notch signaling and subsequently determine the differentiation of HSCs in livers of subjects with CLF. This study provides scientific evidence for improving the treatment of CLF by transplanting BM-MSCs with *Numb* gene editing. Of course, because BM-MSCs with *Numb* knockdown or overexpression were used for transplantation in this study, the method of conditional knockout or knock-in of liver *Numb* must be adopted to clarify the therapeutic value of *Numb* for CLF in the future.

## Conclusions

*Numb* is an important determinant of cell fate. In CLF, *Numb* determines the fate of HSCs, promotes their differentiation into hepatocytes and inhibits their differentiation into BECs by suppressing Notch signaling. Importantly, our results clearly indicated that the transplantation of BM-MSCs with *Numb* overexpression may be a useful new treatment strategy for CLF.

## Supplementary Information

**Additional file 1. **Supplementary Materials.

**Additional file 2. **The original image of the immunoblotting.

## Data Availability

All data generated or analyzed during this study are included in this published article and its Additional files 1, 2.

## Abbreviations

Alb: Albumin
ALP: Alkaline phosphatase
ALT: Alanine aminotransferase
α-SMA: α-Smooth muscle actin
AST: Aspartate aminotransferase
BEC: Biliary epithelial cell
BM-MSCs: Bone marrow-derived mesenchymal stem cells
CLF: Cholestatic liver fibrosis
CLS: C-promoter binding protein-1/suppressor of hairless/Lag1
Col: Collagen
DR: Ductular reaction
GGT: Gamma-glutamyl transferase
HSC: Hepatic stem cell
Hyp: Hydroxyproline
IL: Interleukin
LNX: Ligase Numb protein X
MCP1: Monocyte chemoattractant protein 1
NICD: Notch intracellular domain
PBC: Primary biliary cholangitis
PDGF: Platelet-derived growth factor
PSC: Primary sclerosing cholangitis
RBP-Jκ: Recombination signal binding protein Jκ
TBA: Total bile acid
TBil: Total bilirubin
TGF-β1: Transforming growth factor beta 1
TNF-α: Tumor necrosis factor alpha
