# Supplementary material for: BM-MSCs overexpressing the Numb enhance the therapeutic effect on cholestatic liver fibrosis by inhibiting the ductular reaction
Source: Stem Cell Res Ther. 2023 Mar 20;14:45. doi: 10.1186/s13287-023-03276-w (PMC10029310; doi:10.1186/s13287-023-03276-w)
Supplement: Supplementary file 1 — Additional file 1. Supplementary Materials. [file 13287_2023_3276_MOESM1_ESM.docx]

Supplementary Materials for

***Numb*, a possible therapeutic target for cholestatic liver fibrosis**

Yan-nan Xu, Wen Xu, Xu Zhang, Dan-yang Wang, Xin-rui Zheng, Wei Liu, Jia-mei Chen, Gao-feng Chen, Cheng-hai Liu, Ping Liu, Yong-ping Mu

Correspondence to: ypmu8888@126.com, and Liuliver@vip.sina.com.

**Figure. S1**


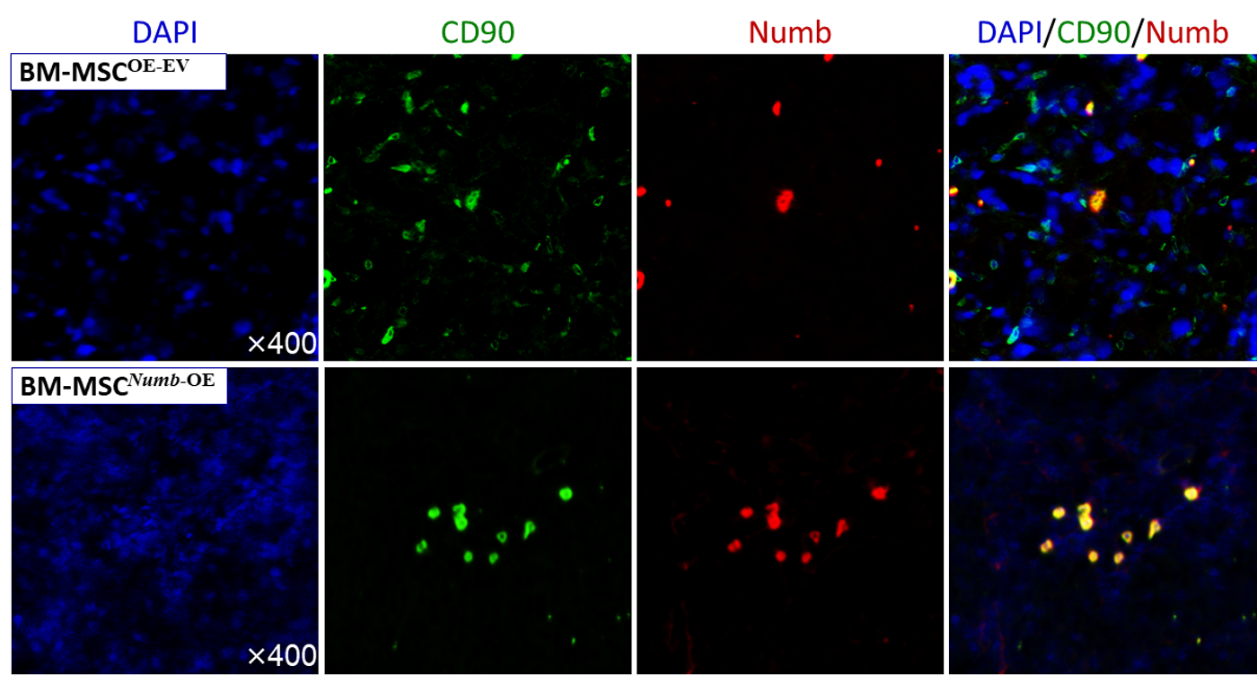


**Fig. S1**. **CD90/Numb immunofluorescence costaining (×400).** We performed double immunofluorescence staining for CD90 (a marker of BM-MSCs) and Numb, and the results showed that CD90 and Numb were still co-expressed in the BM-MSC^OE-EV^ and BM-MSC*^Numb^*^-OE^ groups at the end of the 4^th^ week after BDL, which confirmed that Numb was located in BM-MSCs in the livers of BDL rats.

**Figure. S2**

**
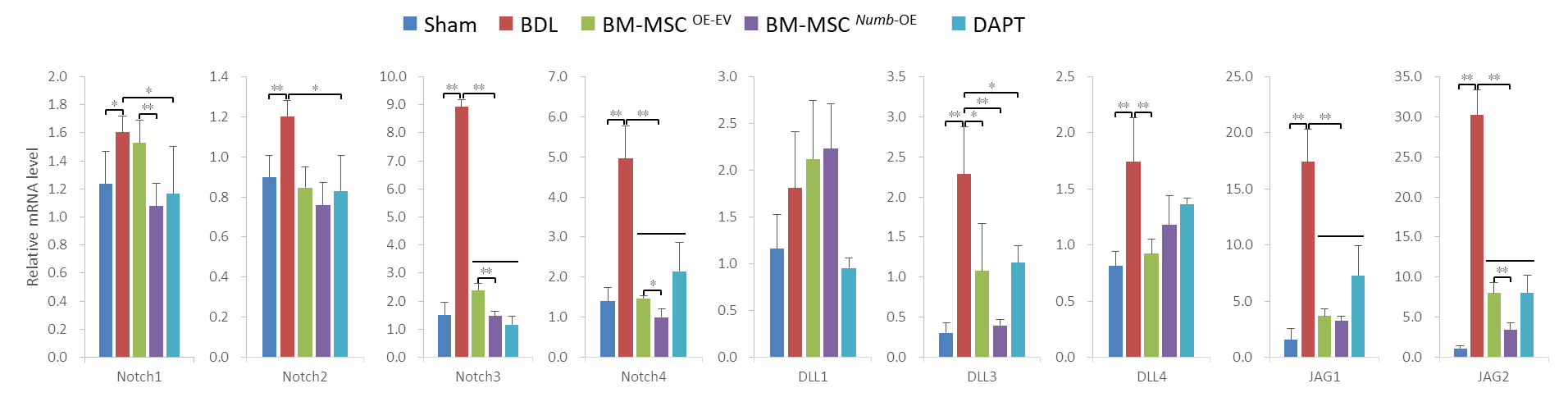
**

**Fig. S2. The expression levels of *Notch-1/-2/-3/-4*, *DLL-1/-3/-4*, and *JAG-1/-2* mRNA.** Sham, sham operation; BDL, common bile duct ligation; BM-MSC^OE-EV^, bone marrow mesenchymal stem cell with empty lentivirus vector corresponding to *Numb* overexpression; BM-MSC*^Numb^*^-OE^, bone marrow mesenchymal stem cell with *Numb* overexpression; DAPT, DAPT injection. * *P* <0.05, ** *P* <0.01.

**Figure. S3**


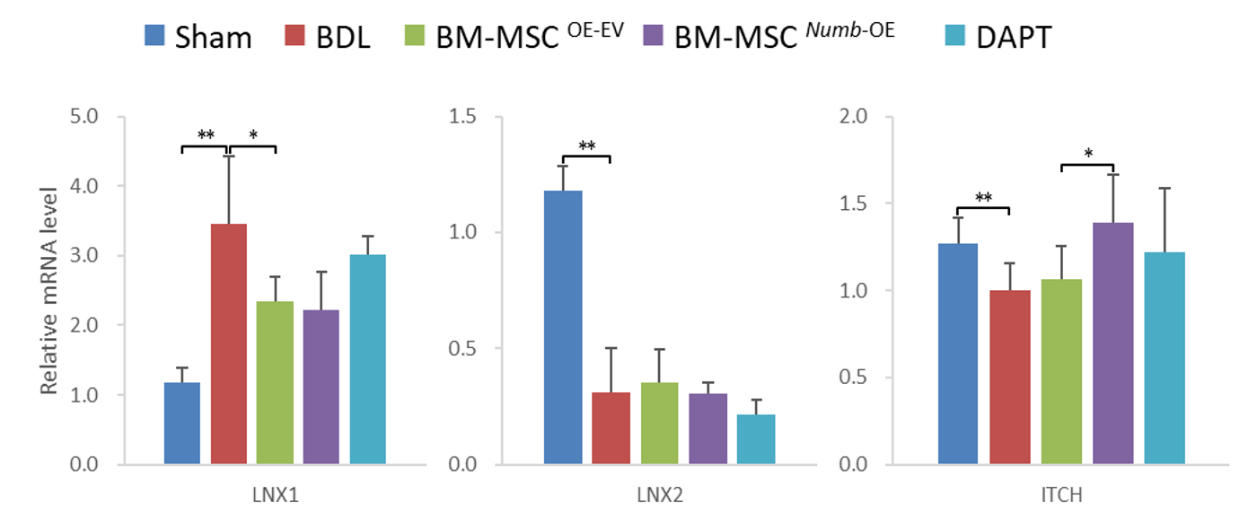


**Fig. S3.** **The expression levels of *LNX1, LNX2* and *ITCH* mRNA.** Sham, sham operation; BDL, common bile duct ligation; BM-MSC^OE-EV^, bone marrow mesenchymal stem cell with empty lentivirus vector corresponding to *Numb* overexpression; BM-MSC*^Numb-^*^OE^, bone marrow mesenchymal stem cell with *Numb* overexpression; DAPT, DAPT injection. * *P* <0.05, ** *P* <0.01.

**Figure. S4**

**
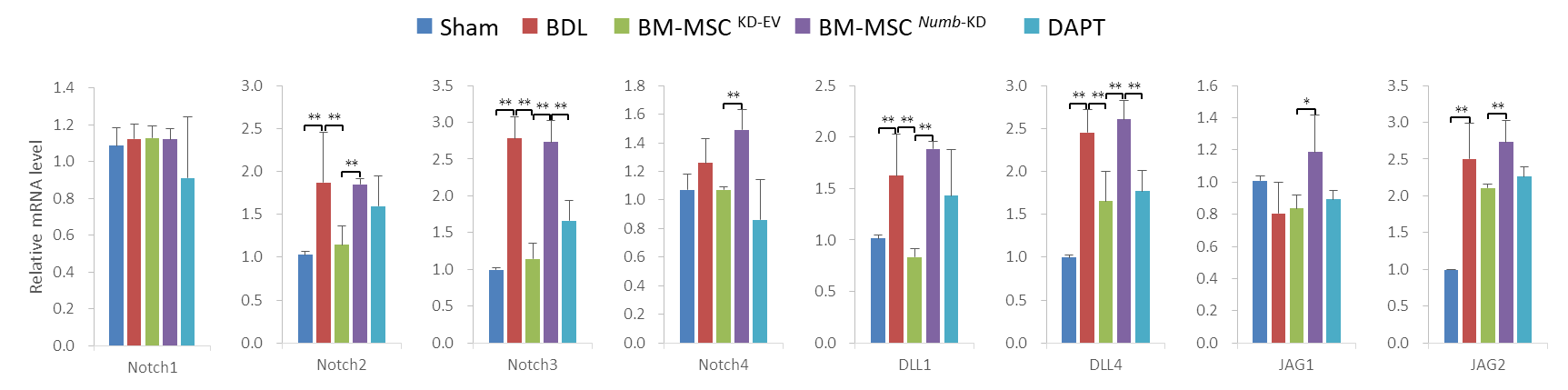
**

**Fig. S4. The expression levels of *Notch-1/-2/-3/-4, JAG-1/-2*, and *DLL-1/-4* mRNA.** Sham, sham operation; BDL, common bile duct ligation; BM-MSC^KD-EV^, bone marrow mesenchymal stem cell with empty lentivirus vector corresponding to *Numb* knockdown; BM-MSC*^Numb-^*^KD^, bone marrow mesenchymal stem cell with *Numb* knockdown; DAPT, DAPT injection. * *P* <0.05, ** *P* <0.01.

**Figure. S5**

**
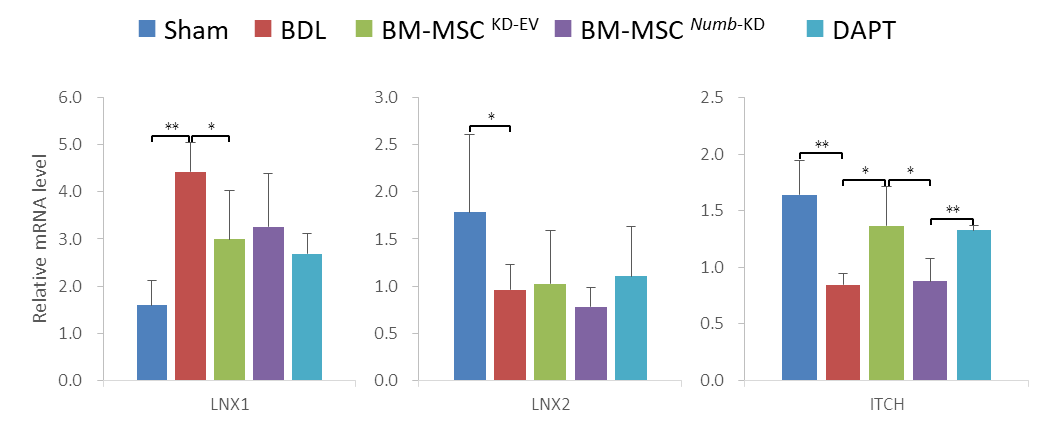
**

**Fig. S5.** **The expression levels of *LNX1, LNX2* and *ITCH* mRNA.** Sham, sham operation; BDL, common bile duct ligation; BM-MSC^KD-EV^, bone marrow mesenchymal stem cell with empty lentivirus vector corresponding to *Numb* knockdown; BM-MSC*^Numb-^*^KD^, bone marrow mesenchymal stem cell with *Numb* knockdown; DAPT, DAPT injection. * *P* <0.05, ** *P* <0.01.

**Supplementary text 1**

**The component sequence of Ubi-MCS-3FLAG-SV40-EGFP-IRES-puromycin**

***Notes:*** *red: Numb NM_133287.1.*

ttttttgttagacgaagcttgggctgcaggtcgactctagaggatccccgggtaccggtcgccaccatgaacaaactacggcagagtttcaggagaaagaaagatgtttacgtcccagaggccagccgtccacatcagtggcagacagatgaagagggagtccgcactggaaagtgcagcttcccagttaagtaccttggccacgtagaggttgatgagtcaagaggaatgcacatctgtgaagatgccgtcaaaagattgaaagctacgggaaagaaagcagtgaaggccgttctgtgggtatcagcagatggactcagagttgtggatgaaaaaactaaggacctcatagttgaccagacaatagaaaaagtttctttctgcgcccccgataggaactttgacagagccttttcttacatatgtcgagatggcaccactcggcgatggatctgtcattgcttcatggctgtcaaagacacgggggaaagactgagccatgccgtgggctgtgcttttgcagcctgtttagagcgtaaacagaagcgggaaaaggagtgtggagtcactgctactttcgatgccagtagaaccacttttacaagagaaggatcattccgtgtcacaactgccacagaacaagctgaaagagaggagatcatgaaacagttgcaagatgccaagaaagctgagacagataagaccgttggtccatcagtggctcctggcaacagtgctccatcgccgtcctctcccacctccccaactctggatcccactgcttctttagagatgaacaatcctcatgctatcccacgccggcatgcaccaattgaacagcttgctcgccaaggctctttccggggatttcctgctcttagccagaagatgtcaccctttaaacgccagctgtccctacgcatcaatgagctgccttccactatgcagaggaagactgatttcccaataaaaaacacagtgcctgaggtggaaggagaggcagaaagcatcagctccctgtgctcccagatcaccagtgccttcagcacaccctgtgaggaccccttctcctctgccccaatgaccaaaccagtgacattagtggcaccacagtctcctgtgttacaagggactgagtggggtcagtcttctggtgctgcctctccaggtctcttccaggctggtcacagacgcactccctctgaggctgaccgttggttagaagaagtatcaaaaagtgtgcgggcccagcagccgcaggcctcagccgcccctctgcagccagttctgcagcctcctccgcccgctgccattgcccctccagcacctcctttccaaggacatgcattcctcacttctcagcctgtgccagtgggtgtggtcccacccctacaaccagcctttgtctctacccagtcctaccctgtggccaatgggatgccctatccagcctctaatgtgcctgtagtgggcatcaccccatcccagatggtagccaatgtgtttggcactgcaggccatcctcaggccactcatccacatcagtccccaagcctggccaagcagcagacattccctcaatatgagacaagtagtgctaccaccagtcccttctttaagccttctgctcagcacctcaatggttctgcagctttcaatggtgtagacaatagcgggctagtctcaggaaacagacctgcacaagtccctccaggcacctgcccagtggatccttttgaggcccagtgggctgcactagaaagcaagcccaagcagcgcaccaacccctctcctaccaaccctttctccagtgatgcacagaaggcatttgaaatagagcttggtatggactacaaggatgacgatgacaaggattacaaagacgacgatgataaggactataaggatgatgacgacaaatgagctagcctgtggaatgtgtgtcagttagggtgtggaaagtccccaggctccccagc
