# Supplementary figures and images for: BM-MSCs overexpressing the Numb enhance the therapeutic effect on cholestatic liver fibrosis by inhibiting the ductular reaction
Source: Stem Cell Res Ther. 2023 Mar 20;14:45. doi: 10.1186/s13287-023-03276-w (PMC10029310; doi:10.1186/s13287-023-03276-w)

**Supplementary Figures**


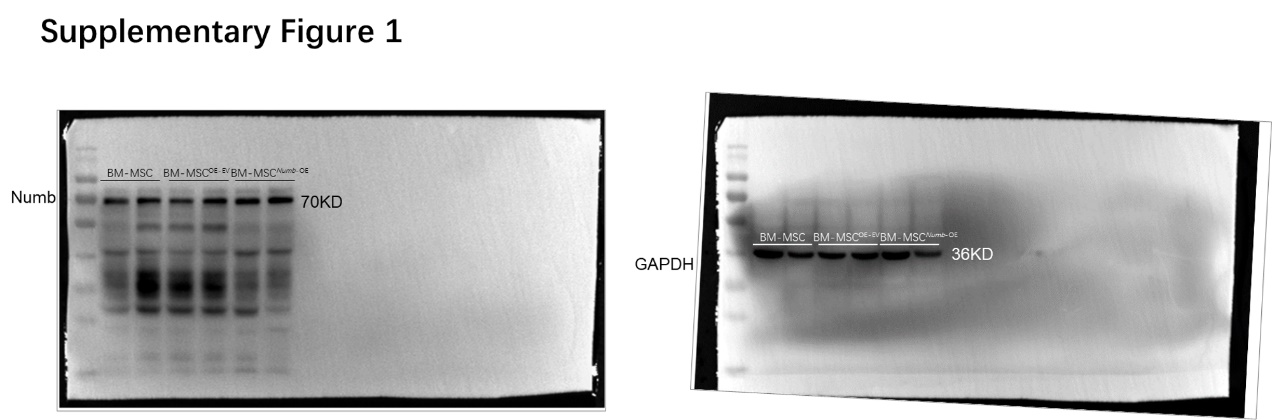


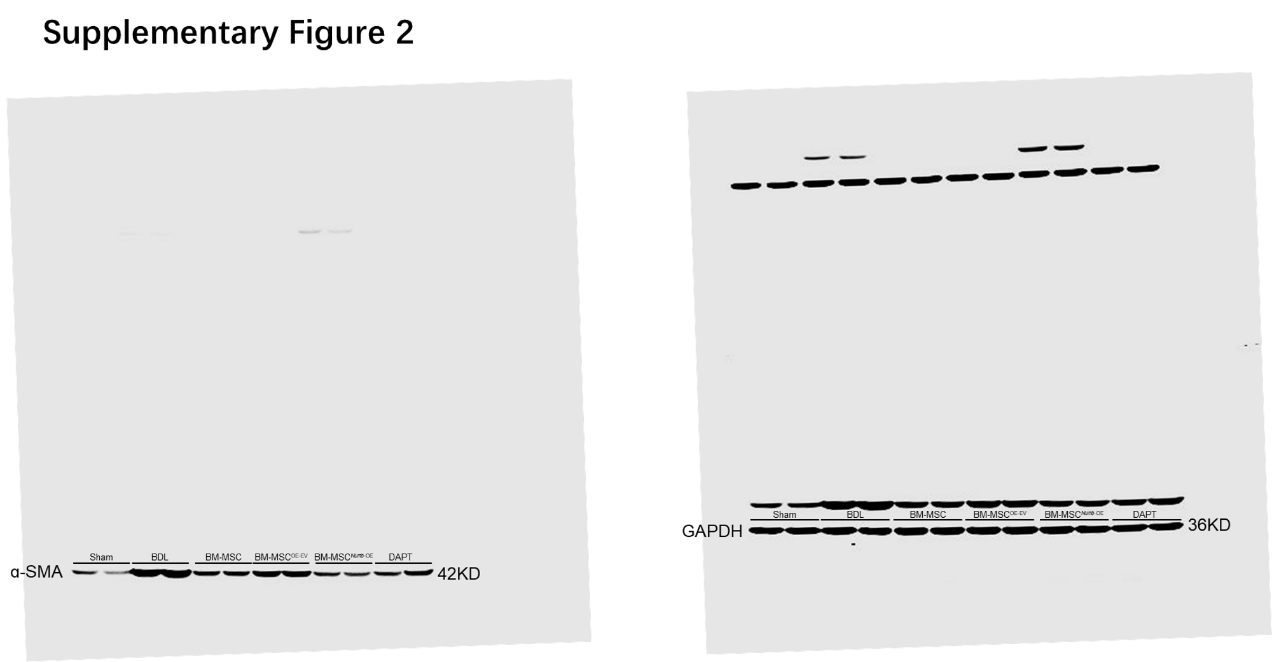


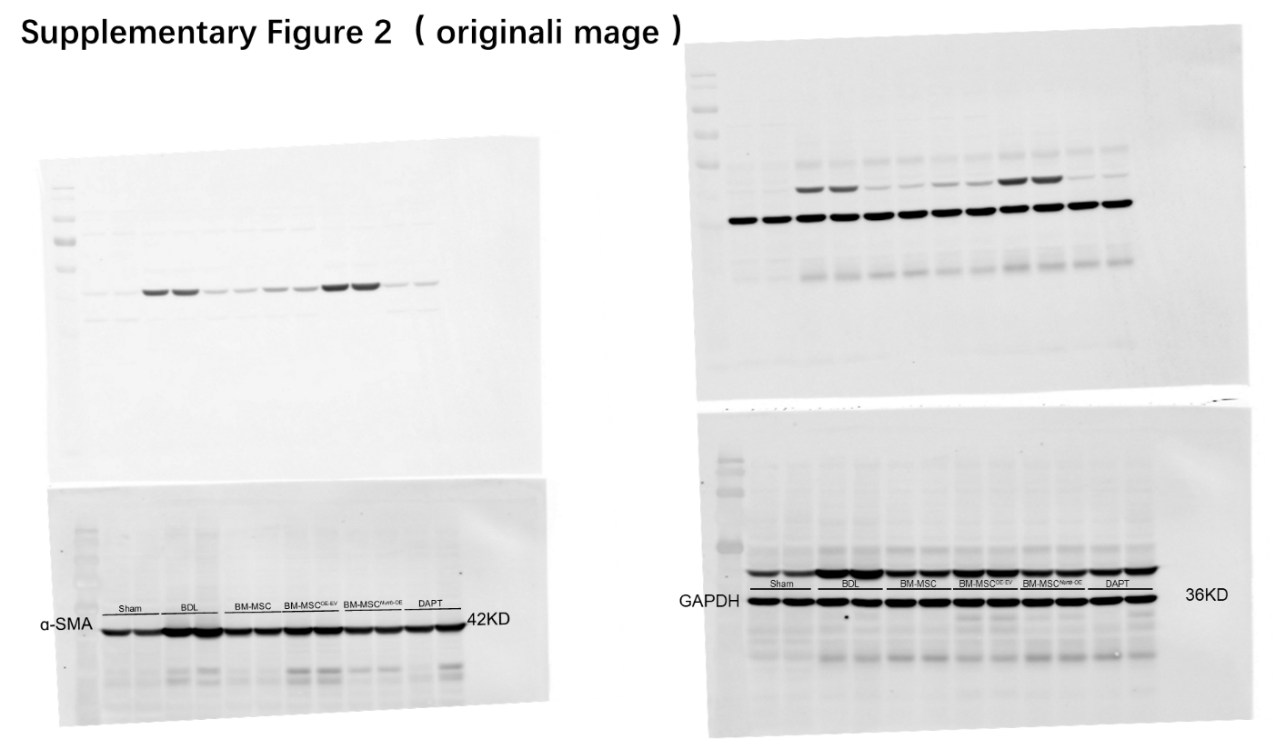


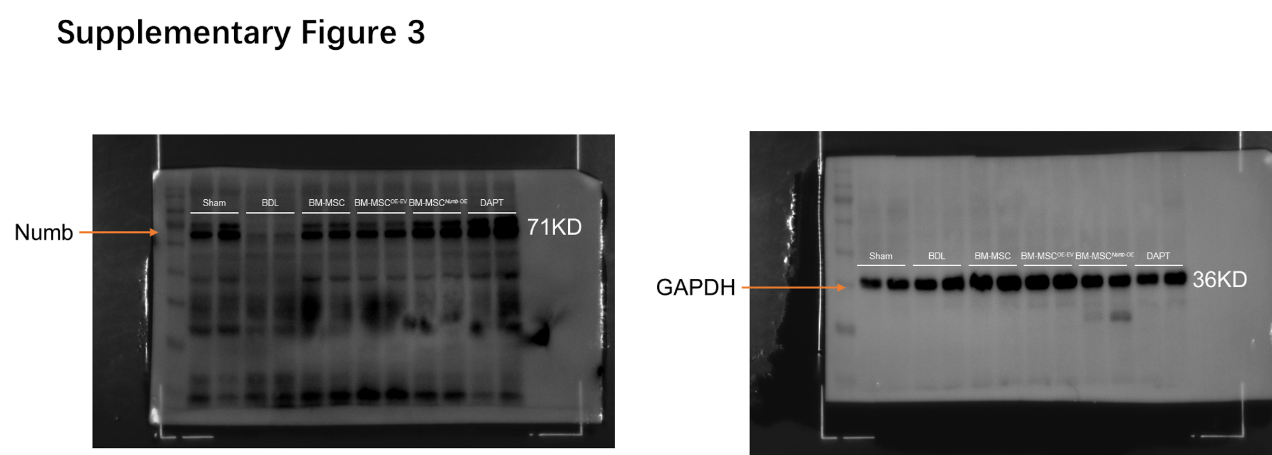


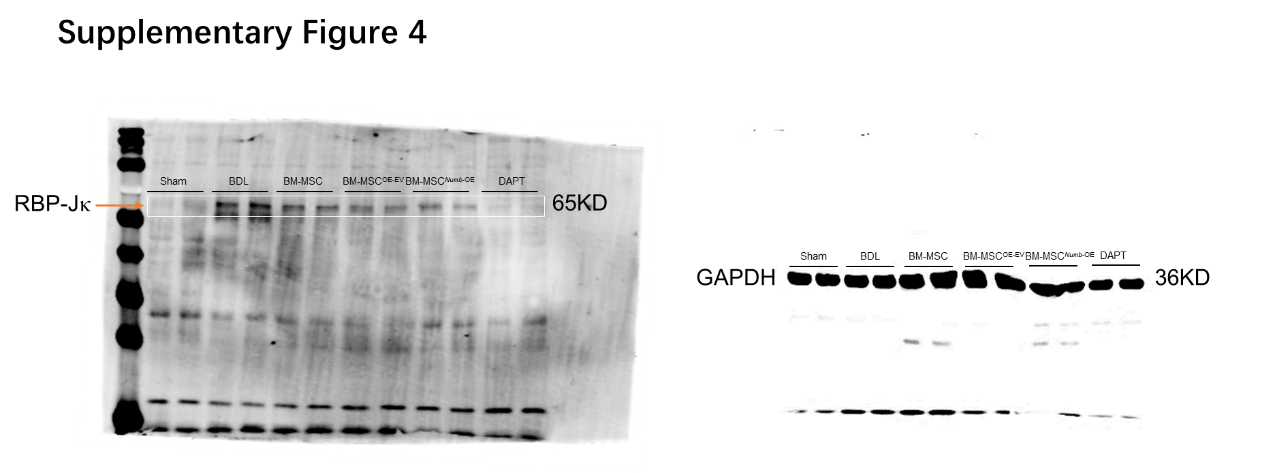


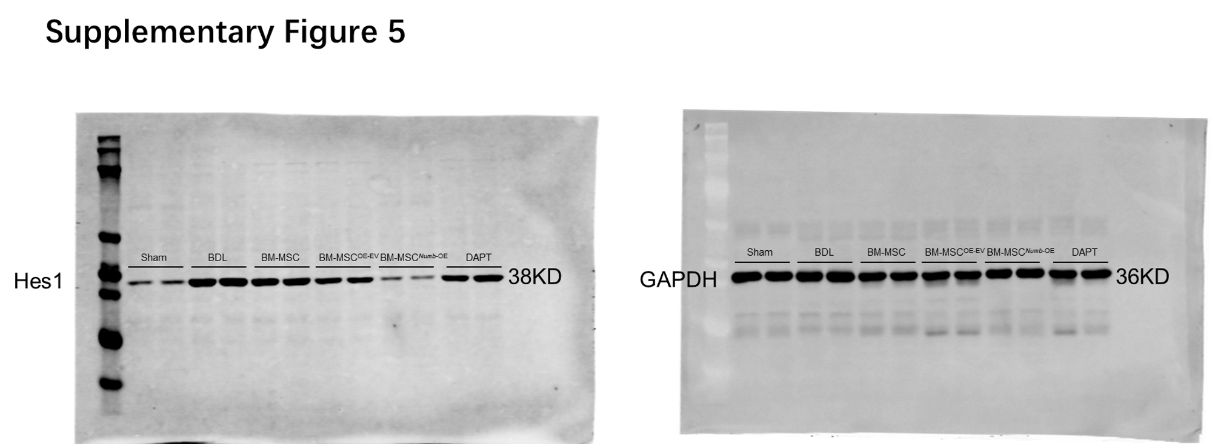


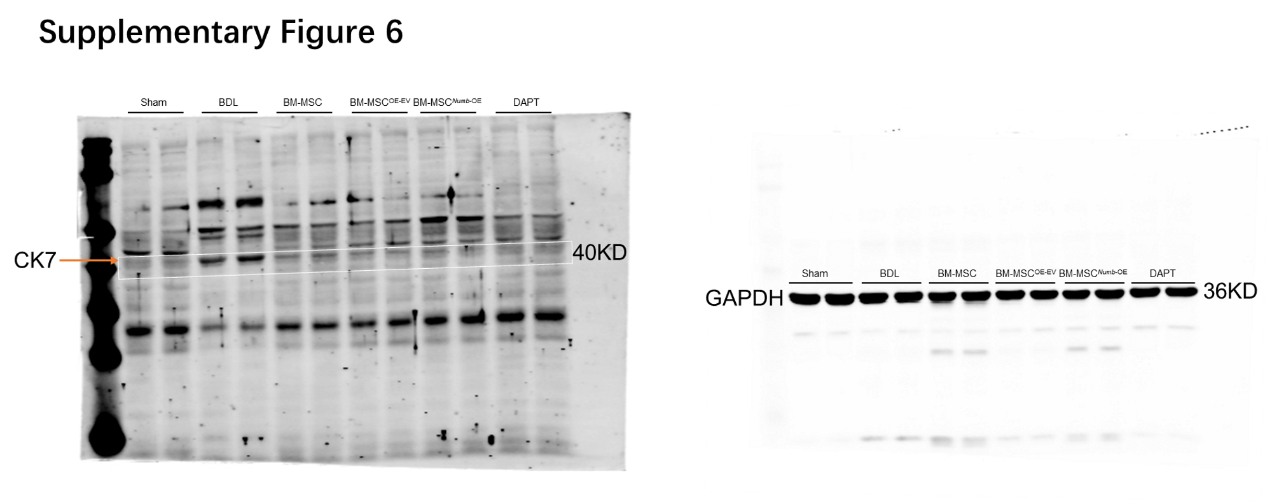


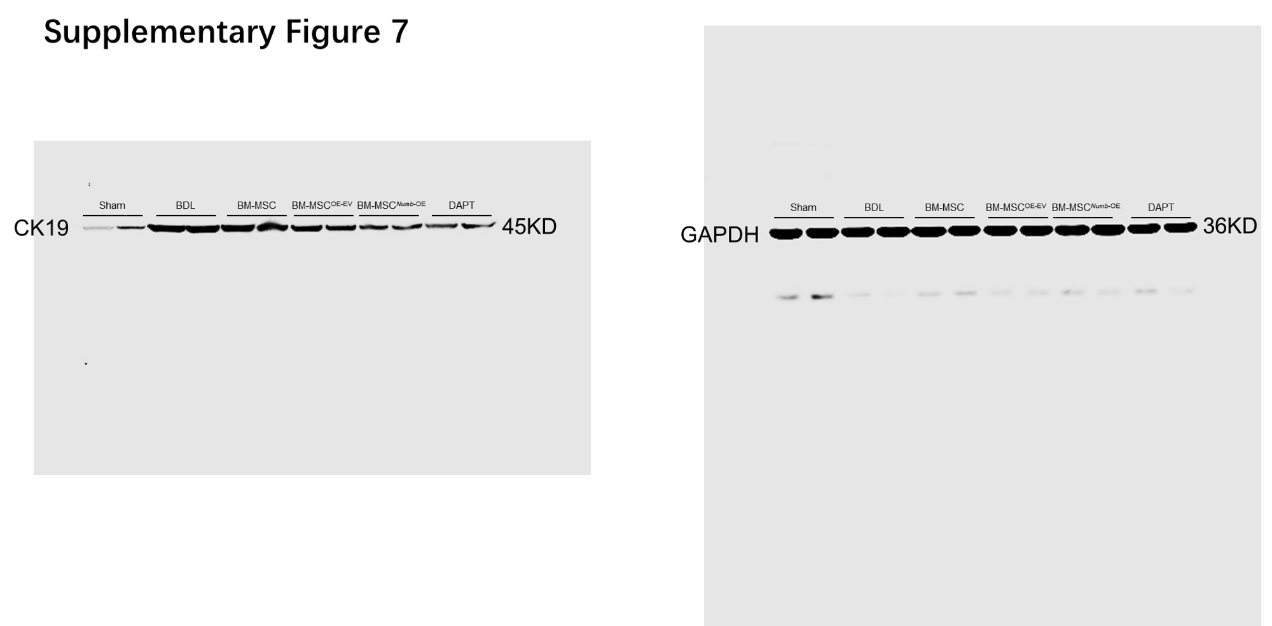


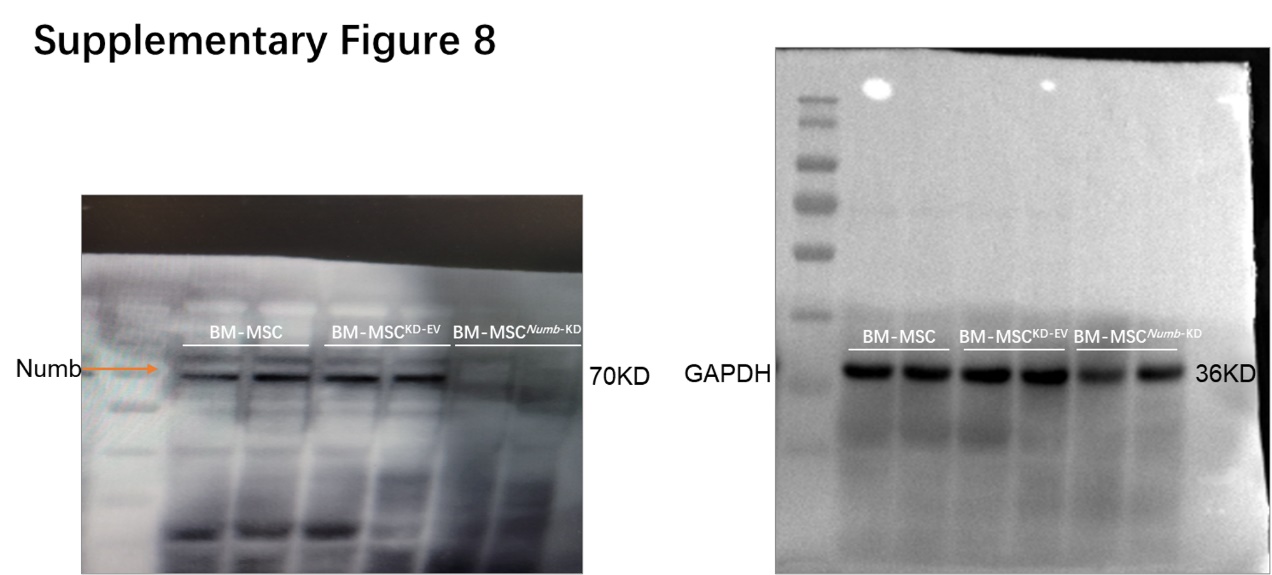


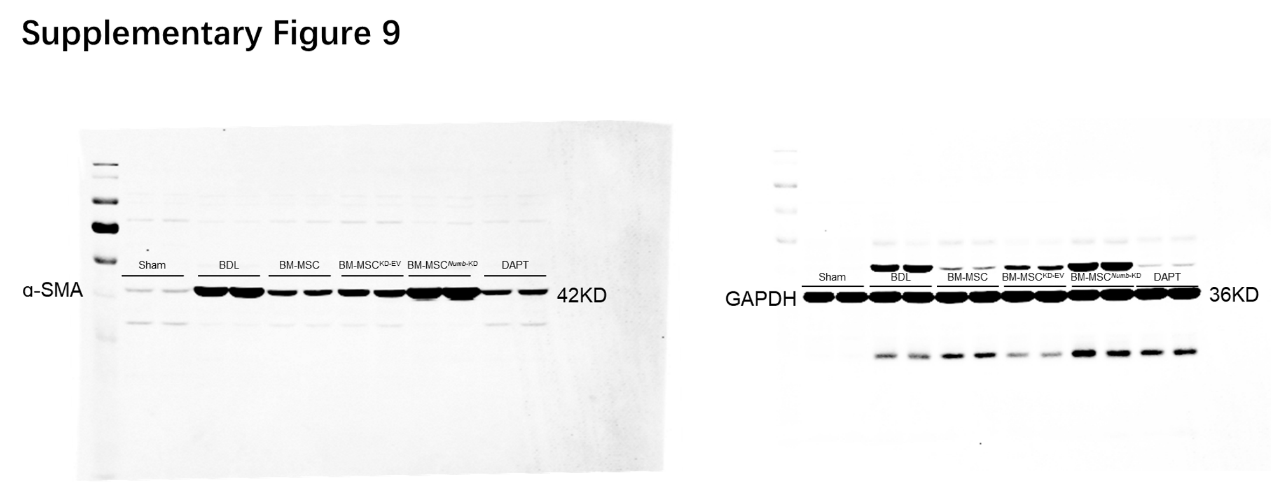


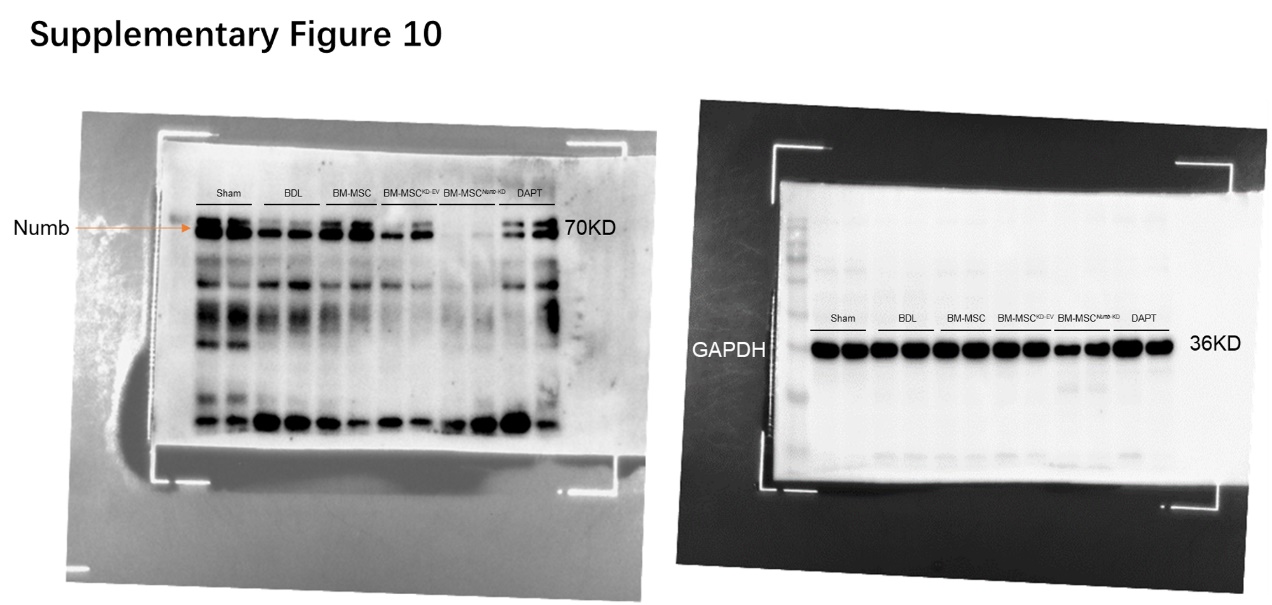


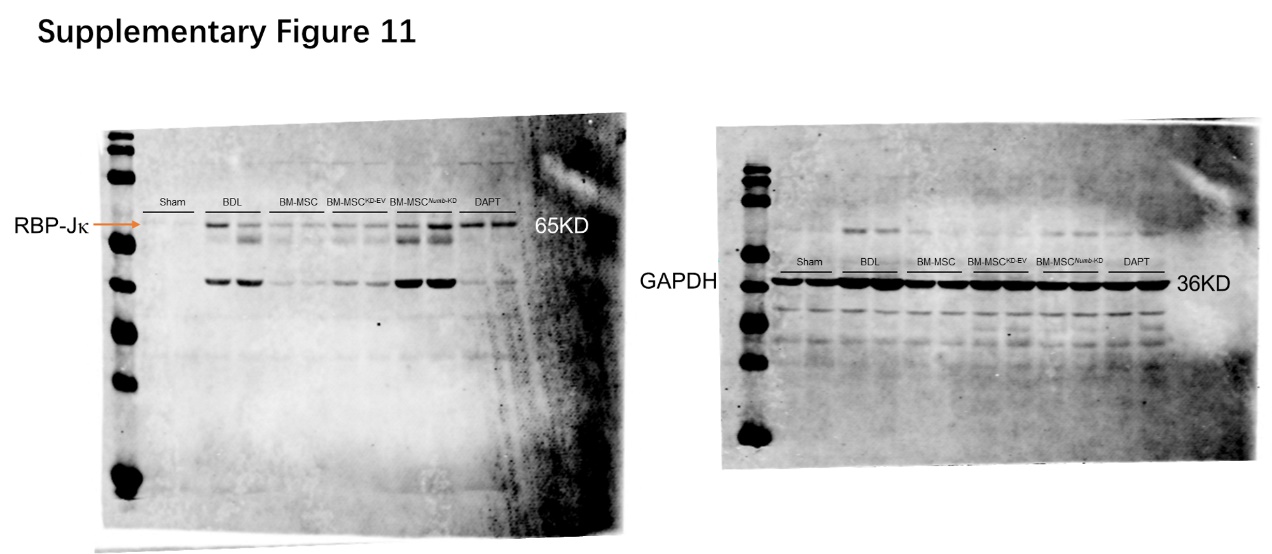


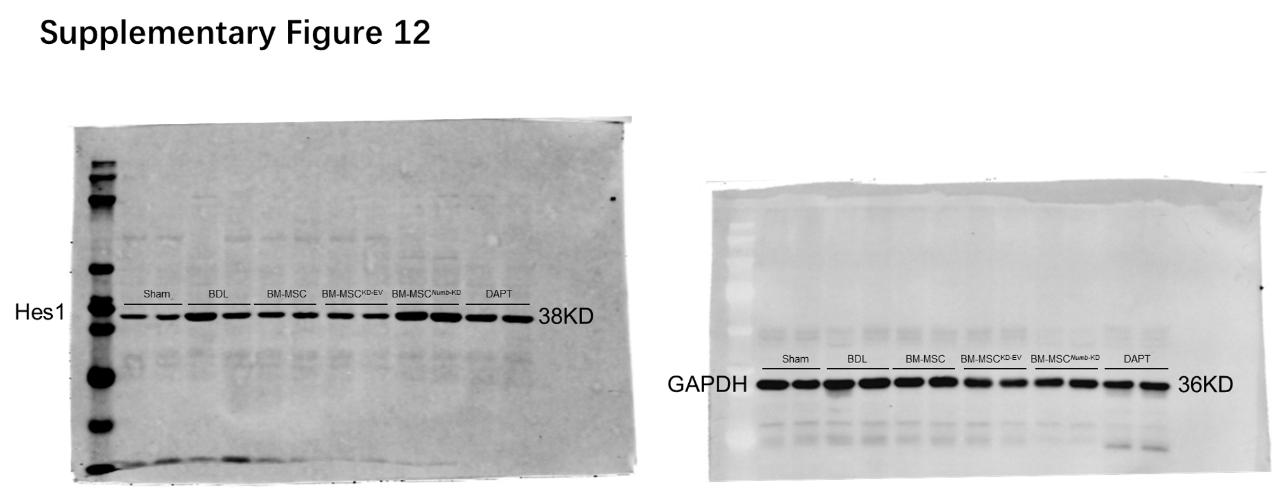


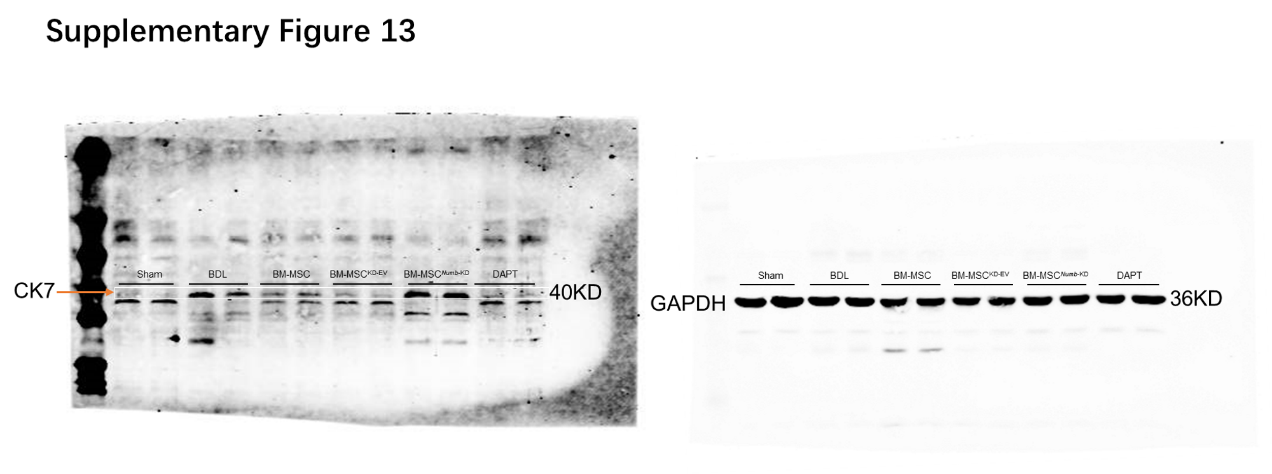


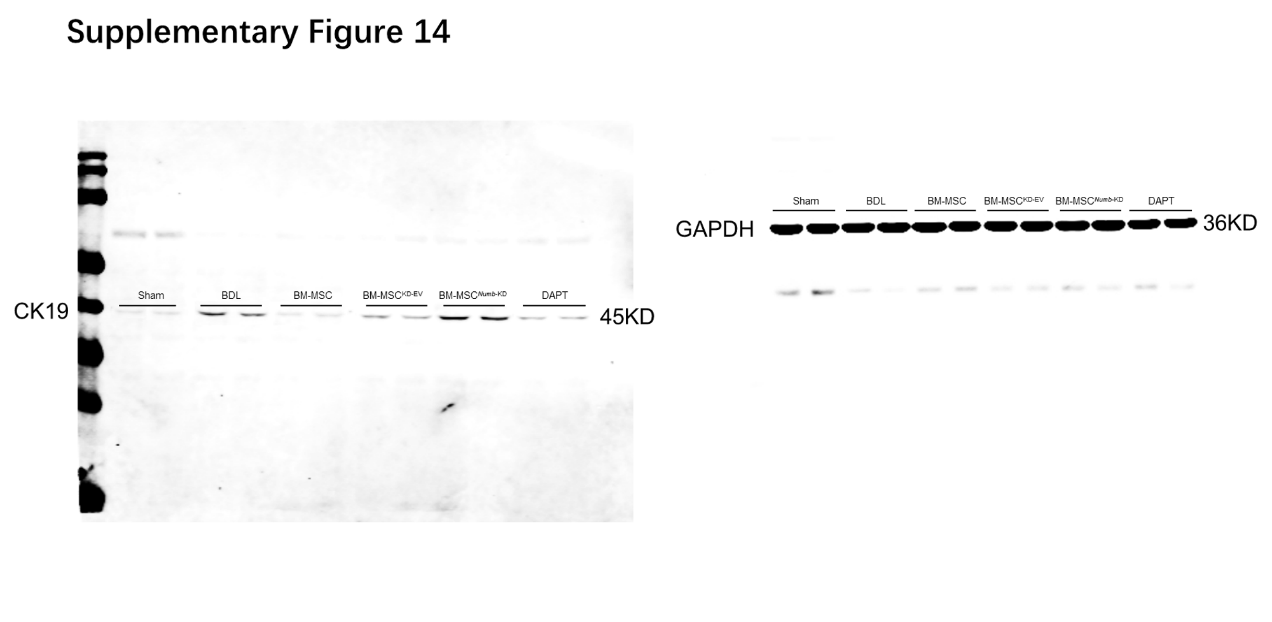


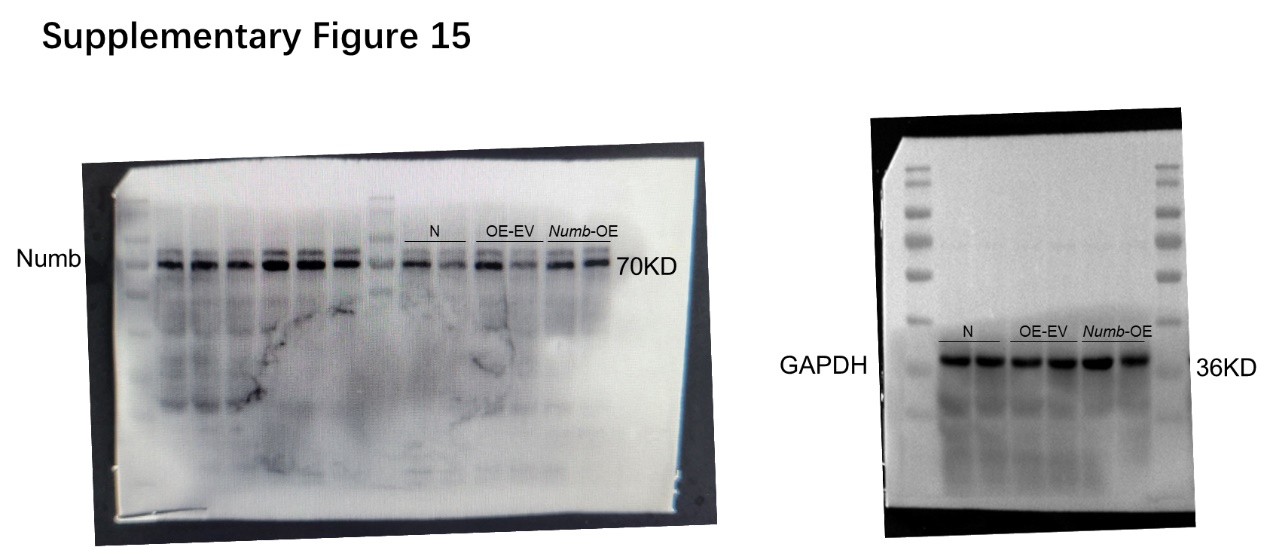


+
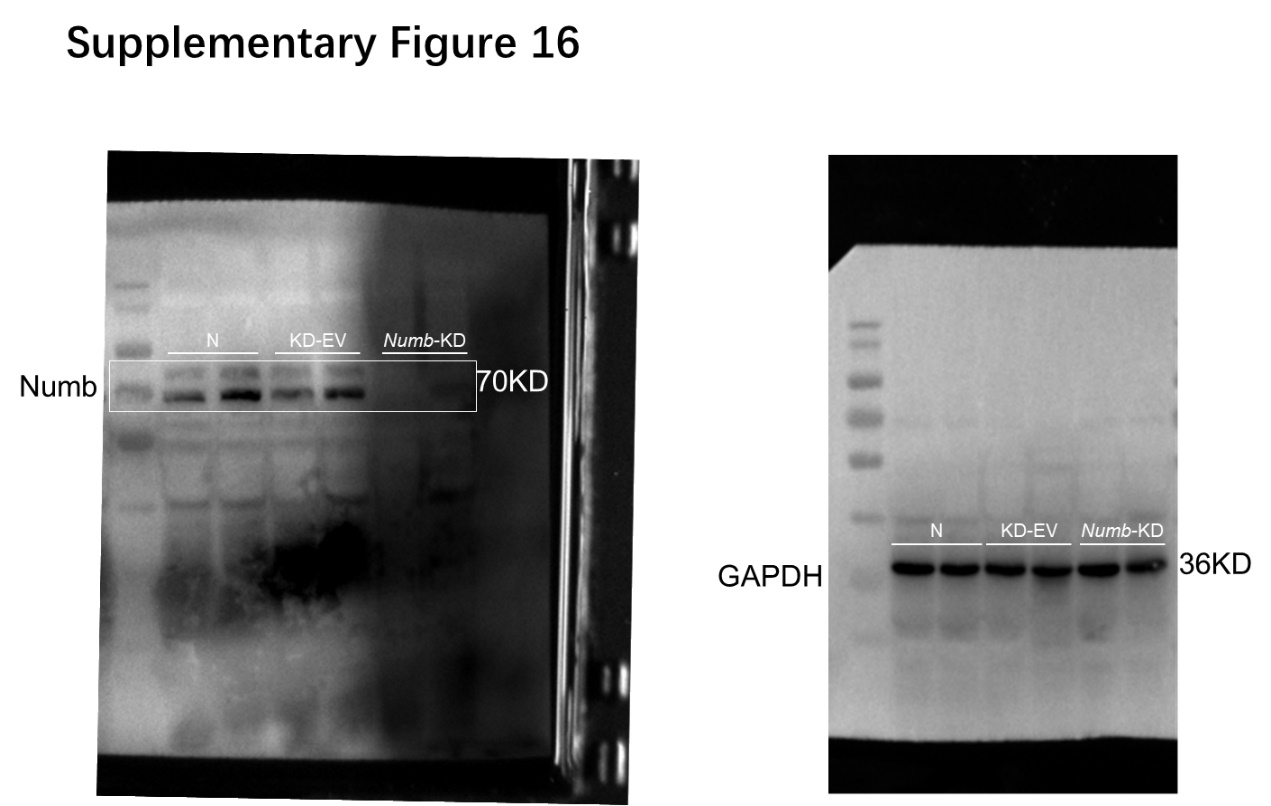

Supplement: Supplementary file 2 — Additional file 2. The original image of the immunoblotting. [file 13287_2023_3276_MOESM2_ESM.docx]
